# Geographical variations and district-level factors associated with COVID-19 mortality in Indonesia: a nationwide ecological study

**DOI:** 10.1186/s12889-023-15015-0

**Published:** 2023-01-14

**Authors:** Henry Surendra, Danarastri Paramita, Nora N. Arista, Annisa I. Putri, Akbar A. Siregar, Evelyn Puspaningrum, Leni Rosylin, Dida Gardera, Montty Girianna, Iqbal R. F. Elyazar

**Affiliations:** 1 Oxford University Clinical Research Unit Indonesia, Faculty of Medicine Universitas Indonesia, Jakarta, Indonesia; 2 Monash University Indonesia, Tangerang Selatan, Indonesia; 3 Komite Pengendalian COVID-19 Dan Pemulihan Ekonomi Nasional, Jakarta, Indonesia; 4 United Nations Development Program, Jakarta, Indonesia; 5 United States Agency of International Development, Jakarta, Indonesia

**Keywords:** COVID-19, Coronavirus, Pandemic, Mortality, Epidemiology, Indonesia

## Abstract

**Background:**

Ensuring health equity, especially for vulnerable populations in less developed settings with poor health system is essential for the current and future global health threats. This study examined geographical variations of COVID-19 mortality and its association with population health characteristics, health care capacity in responding pandemic, and socio-economic characteristics across 514 districts in Indonesia.

**Methods:**

This nationwide ecological study included aggregated data of COVID-19 cases and deaths from all 514 districts in Indonesia, recorded in the National COVID-19 Task Force database, during the first two years of the epidemic, from 1 March 2020 to 27 February 2022. The dependent variable was district-level COVID-19 mortality rate per 100,000 populations. The independent variables include district-level COVID-19 incidence rate, population health, health care capacity, and socio-demographics data from government official sources. We used multivariable ordinal logistic regression to examine factors associated with higher mortality rate.

**Results:**

Of total 5,539,333 reported COVID-19 cases, 148,034 (2.7%) died, and 5,391,299 (97.4%) were recovered. The district-level mortality rate ranged from 0 to 284 deaths per 100,000 populations. The top five districts with the highest mortality rate were Balikpapan (284 deaths per 100,000 populations), Semarang (263), Madiun (254), Magelang (250), and Yogyakarta (247). A higher COVID-19 incidence (coefficient 1.64, 95% CI 1.22 to 1.75), a higher proportion of ≥ 60 years old population (coefficient 0.26, 95% CI 0.06 to 0.46), a higher prevalence of diabetes mellitus (coefficient 0.60, 95% CI 0.37 to 0.84), a lower prevalence of obesity (coefficient -0.32, 95% CI -0.56 to -0.08), a lower number of nurses per population (coefficient -0.27, 95% CI -0.50 to -0.04), a higher number of midwives per population (coefficient 0.32, 95% CI 0.13 to 0.50), and a higher expenditure (coefficient 0.34, 95% CI 0.10 to 0.57) was associated with a higher COVID-19 mortality rate.

**Conclusion:**

COVID-19 mortality rate in Indonesia was highly heterogeneous and associated with higher COVID-19 incidence, different prevalence of pre-existing comorbidity, healthcare capacity in responding the pandemic, and socio-economic characteristics. This study revealed the need of controlling both COVID-19 and those known comorbidities, health capacity strengthening, and better resource allocation to ensure optimal health outcomes for vulnerable population.

**Supplementary Information:**

The online version contains supplementary material available at 10.1186/s12889-023-15015-0.

## Background

The coronavirus disease 2019 (COVID-19) pandemic caused by the spread of severe acute respiratory syndrome coronavirus 2 (SARS-CoV-2) has caused millions of deaths worldwide [1]. As of 23 December 2022, there have been over 6.6 million deaths among over 650 million total confirmed COVID-19 cases reported to the World Health Organization (WHO) [1]. It was estimated that the full impact of the pandemic has been much greater than what is indicated by reported deaths due to COVID-19 [2]. The global impact of the pandemic was heterogeneous and has been associated with different level of pre-existing health systems capacity in managing and responding the pandemic [3, 4].

Ensuring health equity, especially for vulnerable populations in less developed settings with poor health systems is essential for the current COVID-19 pandemic and future global health threats [5–11]. At community-level, it is known that the severity of illness and clinical outcomes can be affected by the concentration of comorbidities in susceptible groups in communities [12–15], and through disparities in access to health care for preventive measures or prompt diagnosis and treatment [15, 16]. Recent findings in US, Chile and Brazil suggested that COVID-19 poor outcome was concentrated in groups with higher socio-demographics and health system vulnerability [15, 17–21]. However, evidence of the geographical variations of COVID-19 impact from low- and middle-income countries (LMIC) where differences in age distribution, comorbidities, access to quality health services, and other factors, may significantly influence mortality risk, are limited.

Indonesia has suffered the highest number of COVID-19 confirmed cases and deaths in Southeast Asia, second only to India in all of Asia [22], at over 6.7 million cases and 160,583 deaths (2.4% case fatality rate (CFR)) up to 29 December 2022 [23]. The first SARS-CoV-2 epidemic wave occurred from 2 March 2020 to 30 April 2021, and a more intense second wave dominated by Delta variant peaked in July 2021 [24], followed by the third wave peaked in February 2022 [23]. The majority of cases and deaths in Indonesia were reported in Java Island, a more developed setting populated by 152 million individuals (56% of the total Indonesia’s population). Recent studies from Indonesia’s capital city of Jakarta suggested that COVID-19 disproportionately affected individuals with older age and pre-existing chronic comorbidities, as well as those areas within Jakarta with lower vaccine coverage, and higher poverty and population density [25, 26]. However, data on the impact of COVID-19 across 514 districts of Indonesia that have different pre-existing burdens of major infectious diseases such as malaria, tuberculosis, HIV and other tropical infections [27], as well as non-communicable diseases like cardiovascular diseases, cancers, chronic pulmonary diseases, diabetes, and others [28], are scarce.

Indonesia is the fourth most populous country (population 270 million) and the LMIC featuring great geographic, cultural and socio-economic diversity across the archipelago. The 2020 Human Development Index (HDI) ranged from 0.32 in Kabupaten Nduga, Papua Province to 0.87 in Kota Yogyakarta, Yogyakarta Province [29]. In addition, substantial proportions of the Indonesian population face barriers in accessing quality health care services due to under-resourced and fragile health systems [27]. A heavily decentralised health systems [30] has resulted in distinct public health capacity across 514 districts of Indonesia. For example, the 2018 Public Health Development Index (PHDI) ranges from 35% in Kabupaten Paniai, Papua Province to 75% in Kabupaten Gianyar, Bali province [31]. That heterogeneity and the large number of COVID-19 cases and deaths provides insights directly relevant to the national public health response to the COVID-19 crisis, and other LMIC settings. Due to the absence of individual-level data of COVID-19 cases and mortality, no study has been done to assess nationwide geographical variations of COVID-19 impacts in Indonesia. In this study, we utilised district-level aggregated data to assess geographical variations of COVID-19 mortality, socio-economics, population health-related conditions, and health care capacity among all 514 districts of Indonesia and how those factors were associated with COVID-19 mortality rate during the first 24 months of the epidemic in Indonesia (March 2020 through February 2022). The study findings will inform decision on health resource allocation and public health interventions to better control COVID-19 pandemic and strengthen health systems resilience in the future.

## Methods

### Study design and participants

This was a nationwide ecological study to assess COVID-19 burden, geographical distributions, and factors associated with mortality rate in all 514 districts in Indonesia. The study analysed aggregated data of individuals diagnosed with COVID-19 based on either rapid antigen diagnostic test (Ag RDT) or polymerase chain reaction (PCR) recorded by the National COVID-19 Task Force from 1 March 2020 to 27 February 2022. In accordance with Indonesia’s national COVID-19 guideline, individuals are categorized as COVID-19 confirmed cases if tested positive by Ag RDT or PCR.

The dependent variable in this study was district-level COVID-19 mortality rate per 100,000 population. The independent variables assessed were COVID-19 incidence rate, proportion of ≥ 60 years old individuals, prevalence of hypertension, prevalence of diabetes mellitus, prevalence of central obesity, prevalence of pneumonia, COVID-19 vaccine coverage for ≥ 60 years old population, number of doctors, nurses, midwives, and hospitals per 10,000 population, expenditure, life expectancy at birth, and length of formal education.

### Data collection

The aggregated data of weekly number of COVID-19 cases, incidence rate per 100,000 population, number of COVID-19 deaths among confirmed cases, and mortality rate per 100,000 population by district from 1 March 2020 to 27 February 2022 were collected from the government official COVID-19 database managed by the National COVID-19 Task Force [32]. District-level data on the number of populations were collected from Statistics Bureau Database available in each province. Data on the number of doctors, nurses, midwives, and hospitals per October 2021 were collected from the Indonesia Ministry of Health records. Data on pre-existing prevalence of hypertension, diabetes mellitus, central obesity, and clinical pneumonia were collected from the latest available National Public Health Development Index (PHDI) 2018 Report [31]. Data on the COVID-19 two-dose vaccine coverage for ≥ 60 years old population by district per 27 February 2022 were collected from the National COVID-19 Vaccination Database [33]. Data on expenditure, life expectancy at birth, and length of formal education were collected from the latest available Human Development Index 2020 Report [29]. The detailed definition and data source of each variable is presented in Table 1.

**Table 1:** Definition of variables and data sources

Variables	Definition	Data sources
**Dependent**		
COVID-19 mortality rate	Number of cumulative deaths among COVID-19 confirmed cases from 1 March 2020 to 27 February 2022	National COVID-19 Task Force [32]
**Independent**		
COVID-19 incidence rate	Number of cumulative COVID-19 confirmed cases from 1 March 2020 to 27 February 2022	National COVID-19 Task Force [32]
COVID-19 vaccine coverage for ≥ 60 years old population	Number of ≥ 60 years old population received two-dose of COVID-19 vaccine per total number of ≥ 60 years old population in 2020	National COVID-19 Vaccination Database [33]
Proportion of ≥ 60 years old individuals	Number of ≥ 60 years old individuals per total number of individuals in 2020	Statistics Bureau Database per province
Prevalence of hypertension	Proportion of individuals aged 15 ≥ years old with systolic blood pressure ≥ 140 mmHg or diastolic blood pressure ≥ 90 mmHg in 2018	Public Health Development Index 2018 [31]
Prevalence of diabetes mellitus	Proportion of individuals aged 15 ≥ years old diagnosed with diabetes mellitus by medical doctor in 2018	Public Health Development Index 2018 [31]
Prevalence of central obesity	Proportion of individuals aged ≥ 15 years old with waist circumference > 90 cm for males and > 80 for females in 2018	Public Health Development Index 2018 [31]
Prevalence of pneumonia	Proportion of individuals all ages diagnosed with clinical pneumonia by either medical doctor/nurse/midwife in the last one month in in 2018	Public Health Development Index 2018 [31]
Doctor per population ratio	Number of doctors per 100,000 population per October 2021	Ministry of Health, Human Resources Data 2021
Nurse per population ratio	Number of nurses per 100,000 population October 2021	Ministry of Health, Human Resources Data 2021
Midwife per population ratio	Number of midwives per 100,000 population October 2021	Ministry of Health, Human Resources Data 2021
Hospital per population ratio	Number of hospitals per 100,000 population October 2021	Ministry of Health, Health Facility Data 2021
Expenditure	Per capita domestic expenditure, measured in 2020	Human Development Index 2020
Life expectancy at birth	The average number of years a newborn is expected to live if mortality patterns at the time of its birth remain constant in the future, measured in 2020	Human Development Index 2020
Length of formal education	The average length of formal education, measured in 2020	Human Development Index 2020

### Statistical analysis

District-level COVID-19 incidence and mortality rate per 100,000 populations, proportion of ≥ 60 years old population, prevalence of hypertension (%), prevalence of diabetes mellitus (%), prevalence of central obesity (%), prevalence of pneumonia (%), COVID-19 vaccine coverage for ≥ 60 years old population (%), number of doctors, nurses, midwives, and hospitals per 100,000 population, expenditure (millions IDR), life expectancy at birth (years), and length of formal education (years) were calculated then categorised into quartiles.

Descriptive statistics included proportions and the chi-squared test to compare district-level characteristics between different quartiles. Spearman’s correlation tests were done to assess correlation between each district-level variable. We used bivariable and multivariable ordinal logistic regression models to determine factors associated with higher mortality rate at district level, expressed as regression coefficient, with 95% confidence intervals (CI). All independent variables with *p*-value < 0.10 in bivariable analysis were included in the multivariable models. Final model selection was informed by likelihood ratio tests.

The proportion of missing data were 2.3% for prevalence of hypertension, 0.6% for diabetes, 1.4% for central obesity, and 7.6% for number of hospitals. Missing-indicator analysis by explanatory variable stratification and by regression analysis identified missing at random, thus justified the exclusion of 47 (9%) districts from the final multivariable model. We set statistical significance at 0.05, and all tests were two-sided. All analyses were done in Stata/IC 15.1 (StataCorp, College Station, TX, USA). Maps showing geographical distribution of COVID-19 incidence and mortality rate were generated using QGIS 3.20 software. This study is reported as per Strengthening the Reporting of Observational Studies in Epidemiology (STROBE) guidelines [32].

## Results

### Cumulative COVID-19 incidence and mortality rate at national-level

**Fig. 1:**
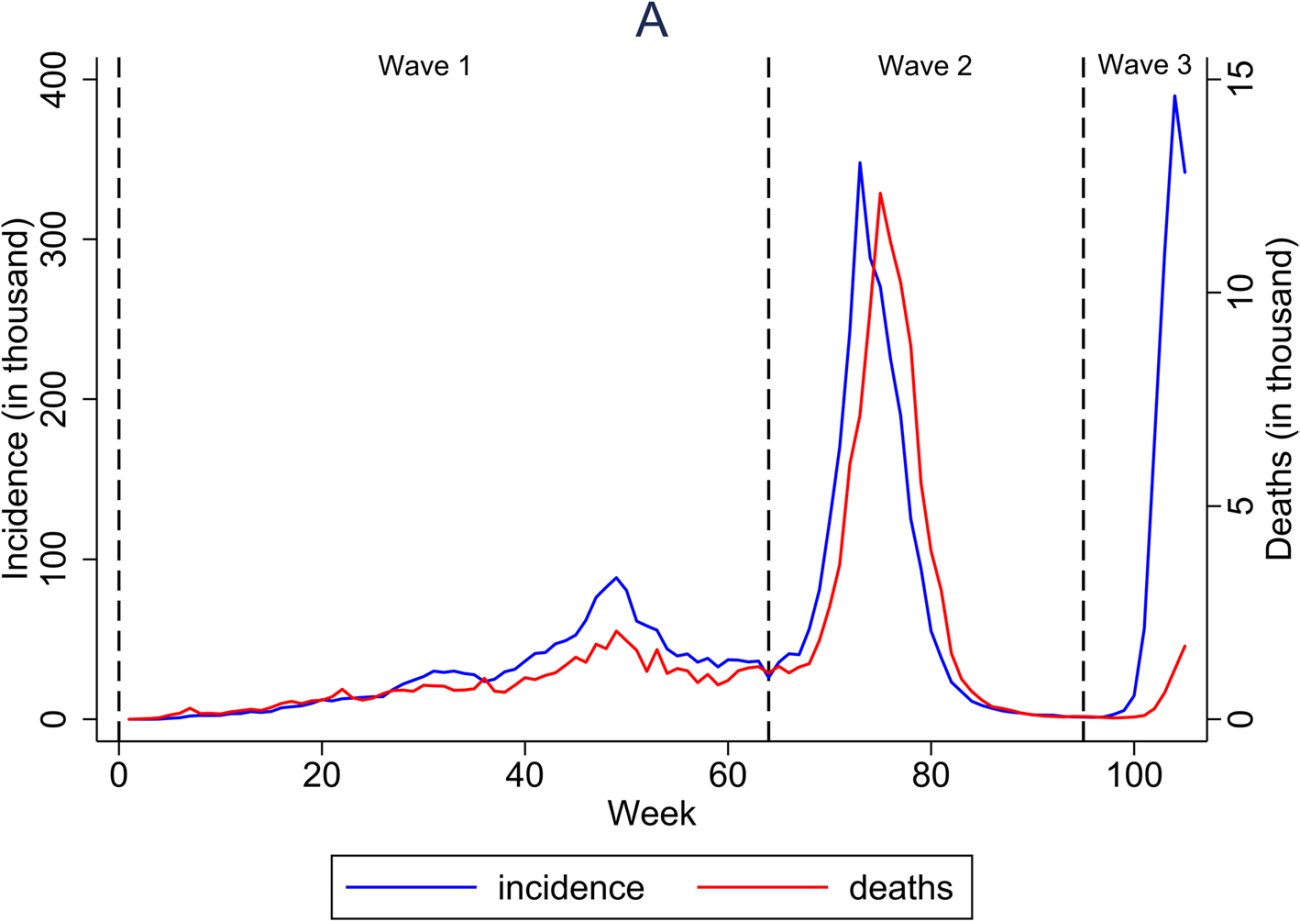
COVID-19 incidence and mortality over the first three epidemic waves in Indonesia between March 2020 to February 2022

**Fig. 2:**
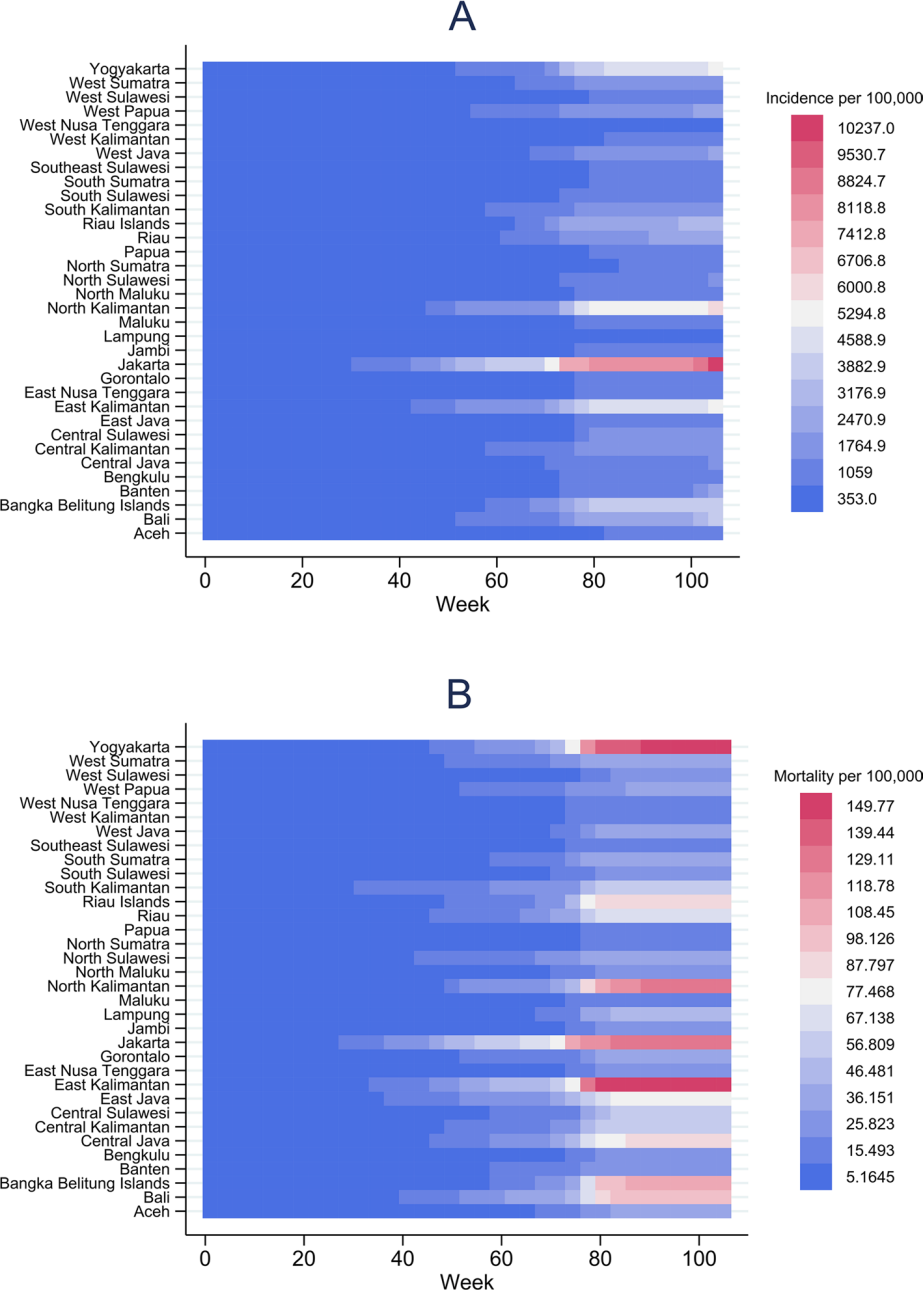
Heatmaps of weekly incidence rate per 100,000 population (**A**) and mortality rate per 100,000 population by province (**B**)

A total of 5,539,333 COVID-19 cases from all 514 districts in Indonesia were recorded on the Indonesia National COVID-19 Database between 1 March 2020 and 27 February 2022. Of those, 148,034 (2.7%) were deceased, and 5,391,299 (97.4%) were recovered. The overall incidence and mortality rates were 2,050 and 55 per 100,000 populations, respectively. Indonesia had experienced three pandemic waves with the highest mortality recorded in week 73^rd^ (12 to 18 July 2021) where cases were predominantly infected by Delta variant (Fig. 1). Overall, the incidence and mortality rate increased over time, with DKI Jakarta being area with consistently high incidence and mortality rate over time (Fig. 2).

### COVID-19 incidence and mortality rate at district-level

**Fig. 3:**
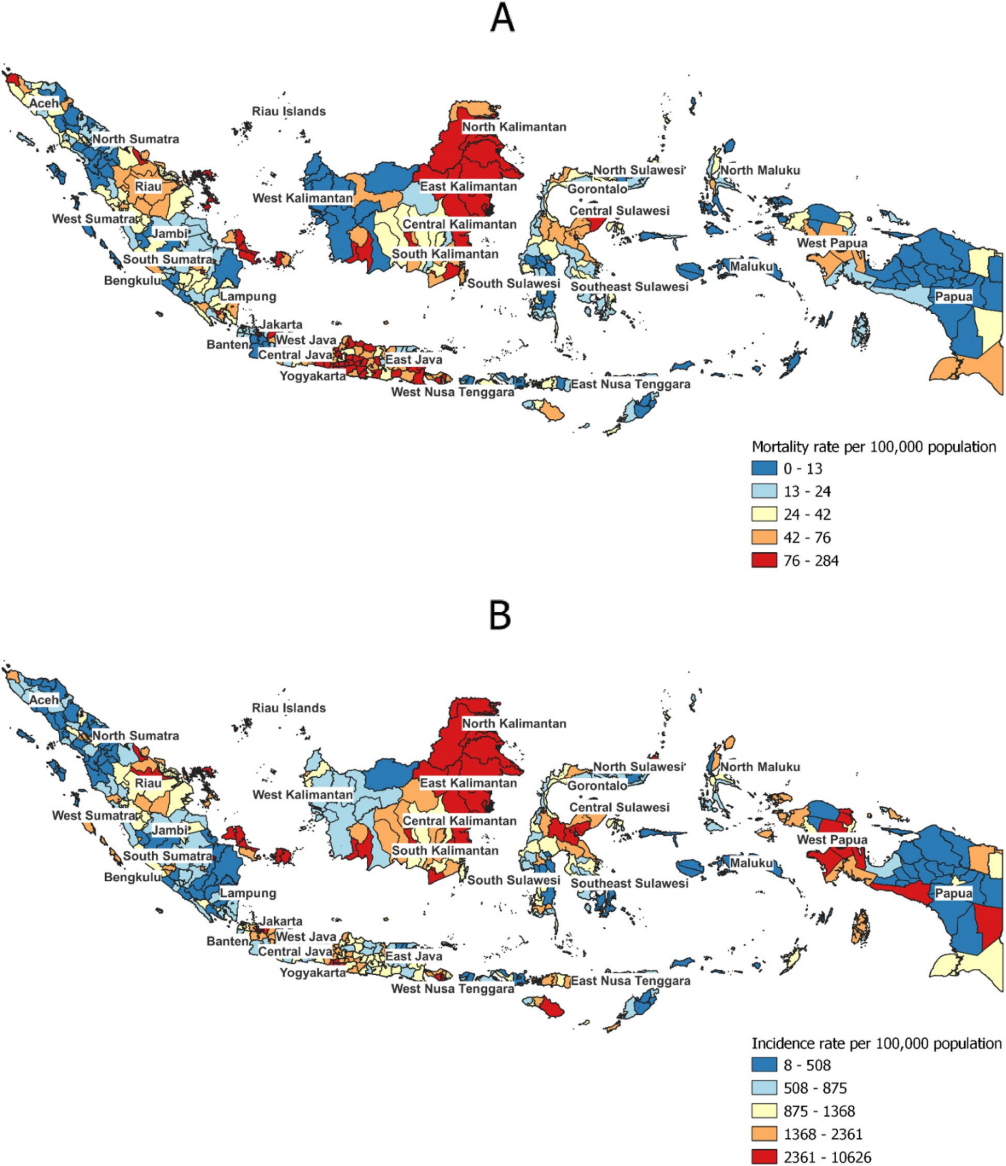
District-level mortality rate (**A**), and incidence rate (**B**) in Indonesia between March 2020 and February 2022

The incidence and mortality rate varied widely by district (Fig. 3). Whilst the incidence rate ranged from 8.1 to 10,626.1 per 100,000 populations (Fig. 3A), the mortality rate ranged from 0 to 283.8 per 100,000 populations (Fig. 3B). Based on Table 2, the top ten highest incidence rate were reported in Kota Jakarta Pusat (10,626 cases per 100,000 populations), Kota Jakarta Selatan (10,308), Kota Bontang (9708), Kota Jakarta Timur (8934), Kabupaten Bulungan (8904), Kota Jakarta Barat (8285), Kota Depok (8229), Kota Jakarta Selatan (8055), Kota Yogyakarta (7806), and Kota Denpasar (7773). The top ten highest mortality rate were reported in Kota Balikpapan (284 deaths per 100,000 populations), Kota Semarang (263), Kota Madiun (254), Kota Magelang (250), Kota Yogyakarta (249), Kota Bontang (247), Kota Surakarta (199), Kota Palangkaraya (184), Kota Tanjungpinang (183), and Kabupaten Berau (178) (Table 3). Summary of incidence and mortality rate in all 514 districts can be found in Supplementary Table 1 and 2.

**Table 2:** Top 10 districts with the highest cumulative COVID-19 incidence rate between March 2020 and February 2022

Rank	District	Province	Total cases	Incidence per 100,000 populations
1	Kota Jakarta Pusat	DKI Jakarta	122,035	10,626
2	Kota Jakarta Selatan	DKI Jakarta	241,535	10,308
3	Kota Bontang	East Kalimantan	17,632	9708
4	Kota Jakarta Timur	DKI Jakarta	284,276	8934
5	Kabupaten Bulungan	North Kalimantan	12,513	8904
6	Kota Jakarta Barat	DKI Jakarta	210,205	8285
7	Kota Depok	West Java	152,874	8229
8	Kota Jakarta Selatan	DKI Jakarta	146,522	8055
9	Kota Yogyakarta	Yogyakarta	32,323	7806
10	Kota Denpasar	Bali	50,468	7773

The full list of 514 districts ranked based on their cumulative incidence rate can be found in Supplementary Table 1

**Table 3:** Top 10 districts with the highest cumulative COVID-19 mortality rate between March 2020 and February 2022

Rank	District	Province	Total deaths	Mortality per 100,000 populations
1	Kota Balikpapan	East Kalimantan	1903	284
2	Kota Semarang	Central Java	4395	263
3	Kota Madiun	East Java	526	254
4	Kota Magelang	Central Java	325	250
5	Kota Yogyakarta	Yogyakarta	1023	249
6	Kota Bontang	East Kalimantan	362	247
7	Kota Surakarta	Central Java	1118	199
8	Kota Palangkaraya	Central Kalimantan	491	184
9	Kota Tanjungpinang	Riau Islands	405	183
10	Kabupaten Berau	East Kalimantan	414	178

The full list of 514 districts ranked based on their cumulative mortality rate can be found in Supplementary Table 2

### COVID-19 burden and other characteristics

The summary of district-level COVID-19 burden and other characteristics associated with COVID-19 mortality rate across 514 districts in Indonesia is presented in Supplementary Table 3. Of 129 districts in the highest mortality rate quartile, 71% had the highest incidence rate. 44% of districts with the highest mortality rate had the highest proportion of ≥ 60 years old population (range 10–20%), and 48% had the highest prevalence of diabetes mellitus (range 3–50%). Regarding health care capacity, 47% of districts with the highest mortality rate had the highest COVID-19 vaccine coverage for ≥ 60 years old population (range 46–86%), and 24% had the lowest number of nurses per 100,000 populations (range 0.5–11). Regarding socio-economic, 57% of districts with the highest mortality rate had the highest expenditure (range IDR 12–24 million) (See Supplementary Table 3 for details).

### Factors associated with higher mortality rate

**Fig. 4:**
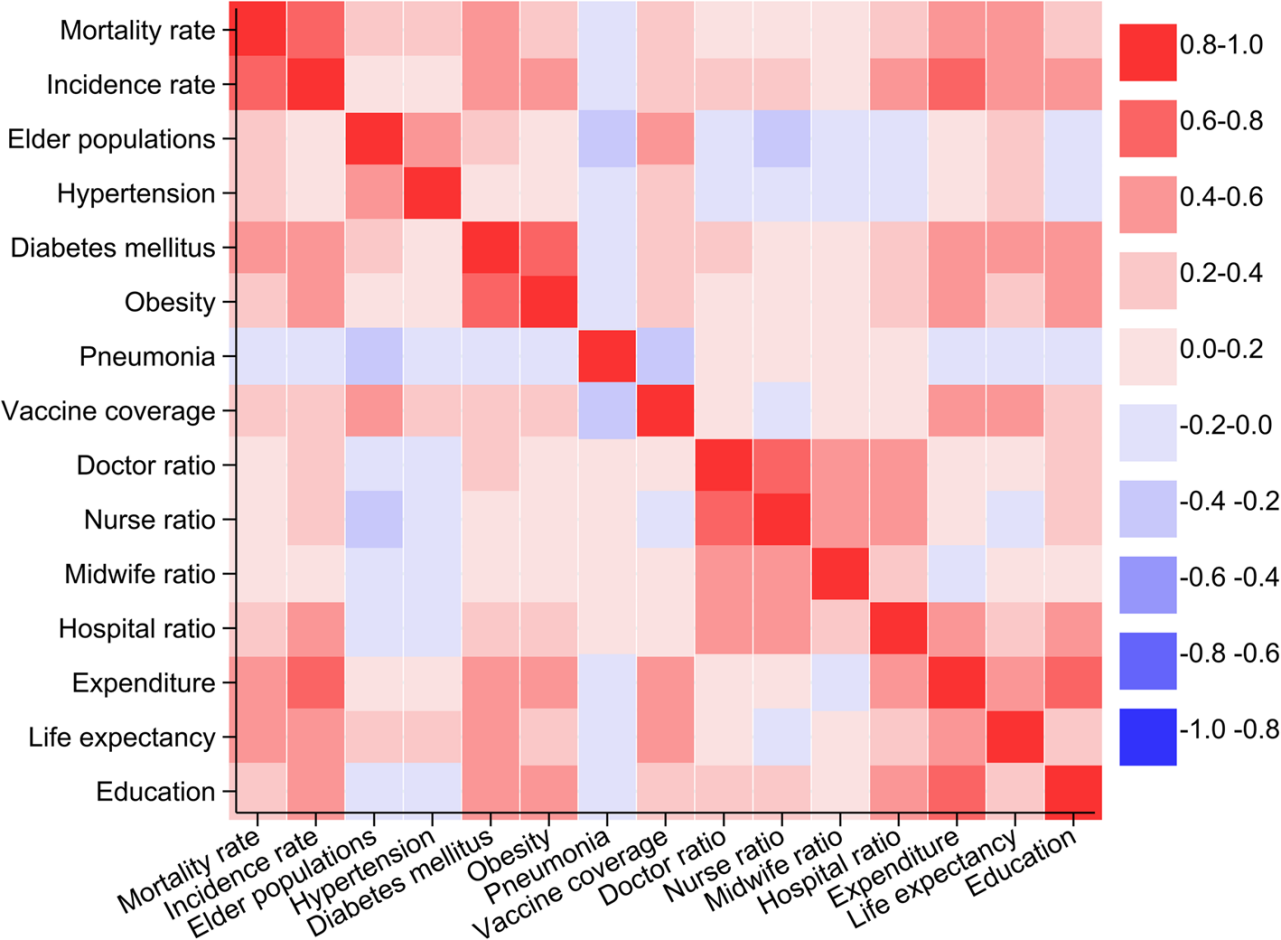
Correlation matrix of COVID-19 burden, prevalence of health-related conditions, vaccine coverage for > 60 years old population, health care capacity, and socio-economic characteristics. Significance level of the correlation can be seen in Supplementary Table 4

In the multivariable model (Table 4), a higher mortality rate was associated with a higher COVID-19 incidence (coefficient 1.64, 95% CI 1.22 to 1.75), a higher proportion of ≥ 60 years old population (coefficient 0.26, 95% CI 0.06 to 0.46), a higher prevalence of diabetes mellitus (coefficient 0.60, 95% CI 0.37 to 0.84), a lower prevalence of central obesity (coefficient -0.32, 95% CI -0.56 to -0.08), a lower number of nurses per population (coefficient -0.27, 95% CI -0.50 to -0.04), a higher number of midwives per population (coefficient 0.32, 95% CI 0.13 to 0.50), and a higher expenditure (coefficient 0.34, 95% CI 0.10 to 0.57). There was no association between COVID-19 mortality rate with prevalence of hypertension, prevalence of pneumonia, vaccine coverage for ≥ 60 years ole population, number of doctors and hospitals per population, life expectancy at birth, and length of formal education (p > 0.05 each). Correlation matrix of COVID-19 burden, prevalence of health-related conditions, vaccine coverage for > 60 years old population, health care capacity, and socio-economic characteristics can be seen in Fig. 4 and Supplementary Table 4.

**Table 4:** District-level factors associated with higher COVID-19 mortality rate in Indonesia

	**Bivariable**		**Multivariable**	
	**Coefficient (95% CI)**	***p* value**	**Coefficient (95% CI)**	**p value**
COVID-19 incidence per 100,000 population	**1.64 (1.44 to 1.84)**	**0.000**	**1.49 (1.22 to 1.75)**	**0.000**
Proportion of ≥ 60 years old population, %	**0.46 (0.32 to 0.61)**	**0.000**	**0.26 (0.06 to 0.46)**	**0.012**
Prevalence of hypertension, %	**0.43 (0.29 to 0.57)**	**0.000**	-0.11 (-0.31 to 0.08)	0.248
Prevalence of diabetes mellitus, %	**0.89 (0.73 to 1.05)**	**0.000**	**0.60 (0.37 to 0.84)**	**0.000**
Prevalence of central obesity, %	**0.51 (0.36 to 0.65)**	**0.000**	**-0.32 (-0.56 to -0.08)**	**0.009**
Prevalence of pneumonia, %	**-0.19 (-0.33 to -0.05)**	**0.007**	-0.01 (-0.19 to 0.16)	0.895
Vaccine coverage for ≥ 60 years old population, %	**0.58 (0.43 to 0.72)**	**0.000**	0.04 (-0.17 to 0.25)	0.709
Doctor per 100,0000 population	**0.37 (0.13 to 0.61)**	**0.003**	0.03 (-0.32 to 0.38)	0.359
Nurse per 100,0000 population	-0.02 (-0.16 to 0.12)	0.760	**-0.27 (-0.50 to -0.04)**	**0.022**
Midwife per 100,0000 population	**0.38 (0.23 to 0.52)**	**0.000**	**0.32 (0.13 to 0.50)**	**0.001**
Hospital per 100,000 population	**0.49 (0.34 to 0.64)**	**0.000**	-0.01 (-0.23 to 0.21)	0.915
Expenditure, thousand IDR	**1.02 (0.85 to 1.18)**	**0.000**	**0.34 (0.10 to 0.57)**	**0.005**
Life expectancy, years	**1.05 (0.89 to 1.22)**	**0.000**	0.23 (-0.00 to 0.46)	0.052
Mean length of formal education, years	**0.51 (0.36 to 0.66)**	**0.000**	-0.17 (-0.40 to 0.07)	0.165

## Discussion

This nationwide ecological study analysed the epidemiological surveillance data of confirmed COVID-19 cases reported from 514 districts in the first 24 months of the SARS-CoV-2 transmission in Indonesia. Indonesia had experienced three pandemic waves with the highest mortality recorded in week 73^rd^ (12 to 18 July 2021) where cases were predominantly infected by Delta variant. The overall COVID-19 case fatality rate was 2.7% (148,034/5,539,333), which equate to a mortality rate of 55 per 100,000 populations. The mortality rate increased over time and was highly heterogeneous over space, with the top ten highest rate seen in Kota Balikpapan, Kota Semarang, Kota Madiun, Kota Magelang, Kota Yogyakarta, Kota Bontang, Kota Surakarta, Kota Palangkaraya, Kota Tanjungpinang, and Kabupaten Berau. A higher district-level mortality rate was associated with a higher COVID-19 incidence, a higher proportion of ≥ 60 years old population, a higher prevalence of diabetes mellitus, a lower prevalence of central obesity, a lower number of nurses per population, a higher number of midwives per population, and a higher expenditure.

Our study described the geographical variations and factors associated with higher COVID-19 mortality in Indonesia. Our findings suggest that the mortality rates tend to be higher in districts located in more developed provinces in Java and Kalimantan Islands (Fig. 2A), and in districts with a higher expenditure. Whilst no previous studies have reported the geographical patterns of COVID-19 mortality in Indonesia, a previous province-level analysis reported DKI Jakarta and other provinces in Java and Kalimantan Islands as the hotspots of cases in the early phase of COVID-19 epidemic in Indonesia [33]. In addition, concordant with the multivariable model suggesting a higher COVID-19 incidence rate as the strongest predictor of a higher COVID-19 mortality rate, the overlapped spatial distribution of high mortality and incidence rate (Fig. 2A-B) further illustrates that districts with a higher incidence rate were more likely to have a higher mortality rate.

Consistent with individual-level risk factors from diverse settings [18, 25, 34, 35], our district-level analysis suggests that higher proportion of elder population and higher prevalence of diabetes mellitus were significantly associated with higher COVID-19 mortality rate in Indonesia. Our analysis reinforces findings from a previous study suggesting that individual-level risk factors such as older age and pre-existing diabetes were associated with an elevated risk of COVID-19 death during the early epidemic phase in DKI Jakarta, Indonesia [25]. These findings indicate the need of controlling both SARS-Cov-2 transmission and non-communicable diseases, especially in areas with high prevalence of diabetes mellitus. Reducing COVID-19 mortality in such settings may necessitate comprehensive and specific interventions such as improving diagnosis and case management of those known non-communicable diseases, community awareness, as well as a sustainable and accessible social security network that may reduce vulnerability of these communities.

Our findings suggesting a higher mortality rate among districts with a higher expenditure was inconsistent with previous findings from a large cohort study in DKI Jakarta, suggesting that risk of mortality was higher for individuals living in areas with higher level of poverty [24]. Whilst previous studies from more developed settings such as in the US [11, 36] and Sweden [37] suggested that higher mortality was associated with lower level of education, our present study found there was no association between mortality rate and level of formal education. A lower educational level might be associated with a lower health literacy that can lead to lower access and understanding of public health information. A tailored risk communication and community engagement strategy targeting population living in districts with lower education level is warranted in addressing health inequity in the future.

Consistent with a previous province-level analysis (*n* = 34) that found the absence of correlation between mortality rate with doctor to population ratio during the first six months of the epidemic [38], our district-level analysis also found the absence of association between mortality rate with doctor to population ratio during the first 24 months of the epidemic. This finding contrasts a previous finding from a higher-income country, reporting an association between higher COVID-19 mortality rate with higher physicians density in France [39]. Nonetheless, our study suggests that a higher mortality rate was associated with a lower number of nurses per population. In Indonesia, the lack of healthcare workers, combined with the high number of infections [40] and deaths among health care workers and doctors [41], especially during the early phase of the epidemic, had weakened the health system resilience. A recent review of Indonesian healthcare system suggested that the currently available medical staffs are insufficient to deal with the pandemic, and that the referral system in Indonesia has been hampered by a shortage of specialists and poorly equipped referral facilities, as well as weak coordination, even before the pandemic [42]. In the context of a heavily decentralized health system such as in Indonesia [30], coordination and prioritisation of available resources and public health intervention will be critical to ensure optimal health outcomes for vulnerable communities, especially for those areas with lower capacity. Short-term human resources strategies can include recruiting trained health care volunteers and redistributing healthcare professionals within province. Moreover, optimising role of residents, fast-tracking nurse training through an accelerated program [43], and recruiting final-year medical students to join the task force within their limits of competence [44] can be potential long-term strategies.

A previous cohort study in DKI Jakarta reported that higher COVID-9-related mortality risk was significantly associated with lower COVID-19 vaccine coverage at subdistrict-level [24]. By contrast, our current study found that there was no association between district-level COVID-9 mortality rate with COVID-19 vaccine coverage among > 60 years old population in Indonesia. This absence of association could possibly be explained by the very low vaccine coverage across the 514 districts. The vaccine coverage was highly varied from 0.03% to 86.20%. The majority of districts (82.3%) still had a vaccine coverage of less than 50% after one year of vaccine roll-out (27 February 2022), thus further highlights the extent of inequity across the country. A previous study from Brazil reported that rapid scaling up of vaccination coverage among elderly Brazilians was associated with significant declines in relative mortality compared with younger individuals, in a setting where the gamma variant predominated [45]. Despite of the current limited available drugs and ineffective vaccine against new emerging variants of SARS-CoV-2 [46], those findings from DKI Jakarta and Brazil, highlight that rapid vaccination roll outs targeting the most vulnerable is crucial in reducing COVID-19-related deaths. As per 29 December 2022, the coverage for two-dose and three-doses COVID-19 vaccination in Indonesia was 86.94% (204,014,895/234,666,020 targeted population) and 74.47% (174,756,560/234,666,020), respectively [47].

This study had several limitations. Firstly, this study was based on routine surveillance data derived from district-level health office case reports. The imperfect contact tracing, testing, and reporting activities could result in underreporting of cases, especially those asymptomatic and mild cases, which could cause an overestimation of case fatality rate and an underestimation of mortality rate per population in this study. Secondly, as this study was based on aggregated data available at district level, our analysis was unable to capture and adjust the effect of important individual-level risk factors. For example, at individual level analysis, the association between a higher COVID-19 mortality with a lower prevalence of central obesity might be confounded by the presence of important effect modifiers variables such as type 2 diabetes, hypertension, and cardiovascular disease [48]. However, we were unable to assess these interactions at our district-level analysis. Therefore, interpretation of the study findings should be restricted to district-level risk factors. Thirdly, the use of data from the 2018 PHDI (the most updated available data) may not reflect the current health systems capacity in responding COVID-19 pandemic in Indonesia. However, the use of these data reflects the existing public health systems capacity in Indonesia prior to the pandemic, which were important indicators of health systems resilience. Finally, previous studies in Indonesia have suggested the association between COVID-19 mortality rate with population density [24] and ratio of hospitals bed [38]. However, these variables were not evaluated in our study, due to limited access to the relevant data. The absence of association for several explanatory variables and the unexplainable association between a higher mortality with a higher number of midwives per population in the present study might be due to the current limitations.

## Conclusions

In conclusion, our analysis suggested that COVID-19 pandemic disproportionately affected districts with high COVID-19 incidence rate, high proportion of elder population, high prevalence of diabetes mellitus, and low nurses to population ratio. These findings indicate that vulnerability to death associated with COVID-19 includes not only elders and comorbid, but also communities living in area with higher COVID-19 incidence and lower health care capacity. These findings may inform decisions on health resource allocation against COVID-19 delivering the greatest possible health dividends by prioritising interventions, including even distribution of essential health care need for the most vulnerable communities in LMIC, especially with decentralised health systems like in Indonesia. Improved diagnosis and case management of those known comorbidities may reduce risk of COVID-19 mortality in these communities. Future nationwide studies incorporating individual and district-level data to assess vulnerability associated with COVID-19-related morbidity and mortality are needed to better comprehend the COVID-19 impact and to better prioritise interventions for the most vulnerable communities.

## Supplementary Information

**Additional file 1.**

## Data Availability

After publication, the datasets used for this study will be made available to others on reasonable requests to the corresponding author, including a detailed research proposal, study objectives and statistical analysis plan.

## Abbreviations

CFR: Case Fatality Rate
CI: Confidence Interval
COVID-19: Coronavirus disease 2019
HDI: Human Development Index
LMIC: Low- and middle-income countries
PHDI: Public Health Development Index
PCR: Polymerase Chain Reaction
Ag RDT: Rapid Antigen Diagnostic Test
SARS-CoV-2: Severe acute respiratory syndrome coronavirus 2
STROBE: Strengthening the Reporting of Observational Studies in Epidemiology
WHO: World Health Organization
