# Supplementary material for: Geographical variations and district-level factors associated with COVID-19 mortality in Indonesia: a nationwide ecological study
Source: BMC Public Health. 2023 Jan 14;23:103. doi: 10.1186/s12889-023-15015-0 (PMC9840537; doi:10.1186/s12889-023-15015-0)
Supplement: Supplementary file 1 — Additional file 1. [file 12889_2023_15015_MOESM1_ESM.docx]

**Geographical variations and district-level factors associated with COVID-19 mortality in Indonesia: a nationwide ecological study**

**Supplementary data**

**Supplementary Table 1. List of districts ranked based on their cumulative COVID-19 incidence rate in Indonesia**

| **Rank** | **District** | **Province** | **Number of cases** | **Incidence per 100,000 populations** |
| --- | --- | --- | --- | --- |
| 1 | Kota Jakarta Pusat | Jakarta | 122035 | 10626 |
| 2 | Kota Jakarta Selatan | Jakarta | 241535 | 10308 |
| 3 | Kota Bontang | East Kalimantan | 17632 | 9708 |
| 4 | Kota Jakarta Timur | Jakarta | 284276 | 8934 |
| 5 | Bulungan | North Kalimantan | 12513 | 8904 |
| 6 | Kota Jakarta Barat | Jakarta | 210205 | 8285 |
| 7 | Kota Depok | West Java | 152874 | 8229 |
| 8 | Kota Jakarta Utara | Jakarta | 146522 | 8055 |
| 9 | Kota Yogyakarta | Yogyakarta | 32323 | 7806 |
| 10 | Kota Denpasar | Bali | 50468 | 7773 |
| 11 | Tana Tidung | North Kalimantan | 1867 | 7732 |
| 12 | Kota Balikpapan | East Kalimantan | 49168 | 7333 |
| 13 | Kutai Barat | East Kalimantan | 11849 | 7223 |
| 14 | Kota Pangkal Pinang | Bangka Belitung Islands | 15526 | 7209 |
| 15 | Badung | Bali | 32014 | 6526 |
| 16 | Kota Bekasi | West Java | 159459 | 6512 |
| 17 | Kota Magelang | Central Java | 8313 | 6390 |
| 18 | Berau | East Kalimantan | 14612 | 6293 |
| 19 | Kota Tarakan | North Kalimantan | 14501 | 6277 |
| 20 | Bantul | Yogyakarta | 58986 | 6213 |
| 21 | Kota Pekanbaru | Riau | 59379 | 6186 |
| 22 | Kota Palangkaraya | Central Kalimantan | 16339 | 6107 |
| 23 | Kota Tangerang Selatan | Banten | 73938 | 5781 |
| 24 | Sleman | Yogyakarta | 60317 | 5608 |
| 25 | Mahakam Ulu | East Kalimantan | 1679 | 5537 |
| 26 | Kepulauan Seribu | Jakarta | 1588 | 5474 |
| 27 | Kota Bogor | West Java | 55519 | 5296 |
| 28 | Belitung | Bangka Belitung Islands | 9107 | 5242 |
| 29 | Manokwari | West Papua | 10007 | 5232 |
| 30 | Kota Banda Aceh | Aceh | 12799 | 5208 |
| 31 | Kota Padang Panjang | West Sumatra | 3000 | 5160 |
| 32 | Kota Tomohon | North Sulawesi | 5056 | 5072 |
| 33 | Kota Tanjung Pinang | Riau Islands | 11097 | 5026 |
| 34 | Kota Surakarta | Central Java | 28114 | 4887 |
| 35 | Kutai Timur | East Kalimantan | 20387 | 4821 |
| 36 | Kota Padang | West Sumatra | 43652 | 4805 |
| 37 | Kota Banjarbaru | South Kalimantan | 11309 | 4763 |
| 38 | Kota Blitar | East Java | 7321 | 4637 |
| 39 | Kota Cirebon | West Java | 15646 | 4597 |
| 40 | Belitung Timur | Bangka Belitung Islands | 5633 | 4485 |
| 41 | Kulon Progo | Yogyakarta | 19887 | 4447 |
| 42 | Kota Madiun | East Java | 9089 | 4394 |
| 43 | Kutai Kartanegara | East Kalimantan | 29911 | 4293 |
| 44 | Kota Jayapura | Papua | 18062 | 4279 |
| 45 | Kota Kupang | East Nusa Tenggara | 18849 | 4277 |
| 46 | Kota Makassar | South Sulawesi | 63220 | 4270 |
| 47 | Kepulauan Anambas | Riau Islands | 1994 | 4257 |
| 48 | Teluk Bintuni | West Papua | 3346 | 4176 |
| 49 | Kota Cilegon | Banten | 17581 | 4153 |
| 50 | Kota Tangerang | Banten | 72593 | 4099 |
| 51 | Bintan | Riau Islands | 6106 | 3899 |
| 52 | Bangka Tengah | Bangka Belitung Islands | 7228 | 3870 |
| 53 | Kota Bukittinggi | West Sumatra | 4767 | 3866 |
| 54 | Nunukan | North Kalimantan | 7033 | 3864 |
| 55 | Kota Semarang | Central Java | 63685 | 3804 |
| 56 | Kota Salatiga | Central Java | 7228 | 3696 |
| 57 | Kota Dumai | Riau | 10823 | 3583 |
| 58 | Kota Mojokerto | East Java | 4903 | 3529 |
| 59 | Malinau | North Kalimantan | 2838 | 3511 |
| 60 | Kota Samarinda | East Kalimantan | 27844 | 3509 |
| 61 | Kota Surabaya | East Java | 103425 | 3506 |
| 62 | Paser | East Kalimantan | 9159 | 3454 |
| 63 | Bangka | Bangka Belitung Islands | 10766 | 3378 |
| 64 | Kota Sukabumi | West Java | 11734 | 3363 |
| 65 | Kota Ambon | Maluku | 11753 | 3361 |
| 66 | Tabanan | Bali | 15128 | 3328 |
| 67 | Kota Solok | West Sumatra | 2451 | 3300 |
| 68 | Kota Manado | North Sulawesi | 14913 | 3151 |
| 69 | Kota Banjarmasin | South Kalimantan | 20889 | 3118 |
| 70 | Bangka Barat | Bangka Belitung Islands | 6205 | 3118 |
| 71 | Kota Palu | Central Sulawesi | 11516 | 3107 |
| 72 | Penajam Paser Utara | East Kalimantan | 5381 | 3098 |
| 73 | Natuna | Riau Islands | 2533 | 3091 |
| 74 | Kota Batam | Riau Islands | 33780 | 3036 |
| 75 | Mimika | Papua | 9382 | 3021 |
| 76 | Kota Malang | East Java | 25874 | 3007 |
| 77 | Kota Bengkulu | Bengkulu | 11111 | 3002 |
| 78 | Kota Bandung | West Java | 74306 | 2996 |
| 79 | Sukamara | Central Kalimantan | 1821 | 2952 |
| 80 | Kota Cimahi | West Java | 16245 | 2934 |
| 81 | Gianyar | Bali | 14673 | 2926 |
| 82 | Bekasi | West Java | 78013 | 2917 |
| 83 | Poso | Central Sulawesi | 7117 | 2875 |
| 84 | Kota Kendari | Southeast Sulawesi | 9417 | 2755 |
| 85 | Kota Banjar | West Java | 5635 | 2740 |
| 86 | Boven Digoel | Papua | 1700 | 2683 |
| 87 | Kota Sorong | West Papua | 7539 | 2680 |
| 88 | Kota Medan | North Sumatra | 67177 | 2673 |
| 89 | Teluk Wondama | West Papua | 1183 | 2671 |
| 90 | Tanah Laut | South Kalimantan | 9203 | 2629 |
| 91 | Fak Fak | West Papua | 2307 | 2620 |
| 92 | Sumba Timur | East Nusa Tenggara | 6306 | 2606 |
| 93 | Morowali Utara | Central Sulawesi | 3175 | 2595 |
| 94 | Kota Palembang | South Sumatra | 41633 | 2571 |
| 95 | Kotawaringin Barat | Central Kalimantan | 6480 | 2552 |
| 96 | Klungkung | Bali | 5457 | 2540 |
| 97 | Kota Tegal | Central Java | 7077 | 2471 |
| 98 | Gunungkidul | Yogyakarta | 18809 | 2443 |
| 99 | Minahasa Utara | North Sulawesi | 5399 | 2432 |
| 100 | Balangan | South Kalimantan | 3150 | 2400 |
| 101 | Kota Gorontalo | Gorontalo | 4797 | 2392 |
| 102 | Kota Tasikmalaya | West Java | 17052 | 2369 |
| 103 | Siak | Riau | 10161 | 2363 |
| 104 | Kota Sawahlunto | West Sumatra | 1578 | 2360 |
| 105 | Bangli | Bali | 6298 | 2354 |
| 106 | Purworejo | Central Java | 18470 | 2328 |
| 107 | Jembrana | Bali | 7613 | 2318 |
| 108 | Lingga | Riau Islands | 2343 | 2317 |
| 109 | Karimun | Riau Islands | 5782 | 2308 |
| 110 | Karawang | West Java | 52362 | 2253 |
| 111 | Kota Mataram | West Nusa Tenggara | 9666 | 2244 |
| 112 | Kota Pontianak | West Kalimantan | 14995 | 2242 |
| 113 | Kota Pekalongan | Central Java | 7026 | 2238 |
| 114 | Kota Probolinggo | East Java | 5365 | 2230 |
| 115 | Bangka Selatan | Bangka Belitung Islands | 4029 | 2223 |
| 116 | Kota Pasuruan | East Java | 4675 | 2216 |
| 117 | Sumba Tengah | East Nusa Tenggara | 1925 | 2187 |
| 118 | Sidoarjo | East Java | 41584 | 2158 |
| 119 | Kota Batu | East Java | 4520 | 2129 |
| 120 | Kota Pare Pare | South Sulawesi | 3156 | 2123 |
| 121 | Klaten | Central Java | 28025 | 2117 |
| 122 | Kota Payakumbuh | West Sumatra | 2916 | 2096 |
| 123 | Kepulauan Mentawai | West Sumatra | 1850 | 2082 |
| 124 | Jayapura | Papua | 3536 | 2071 |
| 125 | Karanganyar | Central Java | 18693 | 2013 |
| 126 | Banggai | Central Sulawesi | 7355 | 2000 |
| 127 | Kota Pematangsiantar | North Sumatra | 5434 | 1998 |
| 128 | Tanah Bumbu | South Kalimantan | 6381 | 1991 |
| 129 | Kota Singkawang | West Kalimantan | 4665 | 1965 |
| 130 | Murung Raya | Central Kalimantan | 2150 | 1955 |
| 131 | Kota Metro | Lampung | 3338 | 1954 |
| 132 | Kota Jambi | Jambi | 11879 | 1927 |
| 133 | Kota Bitung | North Sulawesi | 4351 | 1925 |
| 134 | Kudus | Central Java | 16473 | 1915 |
| 135 | Kota Serang | Banten | 12420 | 1904 |
| 136 | Luwu Timur | South Sulawesi | 5674 | 1889 |
| 137 | Barito Timur | Central Kalimantan | 2148 | 1885 |
| 138 | Tangerang | Banten | 52573 | 1881 |
| 139 | Morowali | Central Sulawesi | 2735 | 1857 |
| 140 | Semarang | Central Java | 19060 | 1843 |
| 141 | Kota Palopo | South Sulawesi | 3258 | 1791 |
| 142 | Bogor | West Java | 83644 | 1774 |
| 143 | Kota Kediri | East Java | 5206 | 1765 |
| 144 | Kota Pariaman | West Sumatra | 1634 | 1742 |
| 145 | Kuantan Singingi | Riau | 5804 | 1742 |
| 146 | Kendal | Central Java | 17612 | 1740 |
| 147 | Sigi | Central Sulawesi | 4326 | 1711 |
| 148 | Magetan | East Java | 11957 | 1710 |
| 149 | Gunung Mas | Central Kalimantan | 2298 | 1696 |
| 150 | Buol | Central Sulawesi | 2387 | 1691 |
| 151 | Bengkalis | Riau | 9363 | 1690 |
| 152 | Kepulauan Aru | Maluku | 1787 | 1690 |
| 153 | Biak Numfor | Papua | 2451 | 1685 |
| 154 | Minahasa | North Sulawesi | 5762 | 1684 |
| 155 | Raja Ampat | West Papua | 1104 | 1674 |
| 156 | Jepara | Central Java | 19913 | 1668 |
| 157 | Lembata | East Nusa Tenggara | 2318 | 1666 |
| 158 | Kota Bau Bau | Southeast Sulawesi | 2628 | 1663 |
| 159 | Banyumas | Central Java | 29873 | 1660 |
| 160 | Ende | East Nusa Tenggara | 4539 | 1647 |
| 161 | Sukoharjo | Central Java | 14895 | 1643 |
| 162 | Purwakarta | West Java | 15472 | 1629 |
| 163 | Wonosobo | Central Java | 14413 | 1625 |
| 164 | Barito Kuala | South Kalimantan | 5082 | 1607 |
| 165 | Temanggung | Central Java | 12580 | 1590 |
| 166 | Kota Bima | West Nusa Tenggara | 2360 | 1579 |
| 167 | Tana Toraja | South Sulawesi | 4528 | 1576 |
| 168 | Aceh Besar | Aceh | 6202 | 1574 |
| 169 | Tojo Una Una | Central Sulawesi | 2587 | 1572 |
| 170 | Sragen | Central Java | 15685 | 1572 |
| 171 | Gresik | East Java | 19578 | 1535 |
| 172 | Indragiri Hulu | Riau | 6594 | 1533 |
| 173 | Buleleng | Bali | 12607 | 1531 |
| 174 | Pulau Morotai | North Maluku | 1140 | 1529 |
| 175 | Manggarai Barat | East Nusa Tenggara | 3998 | 1521 |
| 176 | Kota Lubuk Linggau | South Sumatra | 3431 | 1509 |
| 177 | Hulu Sungai Utara | South Kalimantan | 3437 | 1492 |
| 178 | Sinjai | South Sulawesi | 3879 | 1485 |
| 179 | Tapin | South Kalimantan | 2789 | 1481 |
| 180 | Manggarai | East Nusa Tenggara | 4784 | 1478 |
| 181 | Samosir | North Sumatra | 2044 | 1462 |
| 182 | Barito Selatan | Central Kalimantan | 1895 | 1460 |
| 183 | Kota Prabumulih | South Sumatra | 2855 | 1453 |
| 184 | Pacitan | East Java | 8651 | 1450 |
| 185 | Ngada | East Nusa Tenggara | 2446 | 1448 |
| 186 | Kota Sungai Penuh | Jambi | 1460 | 1444 |
| 187 | Minahasa Tenggara | North Sulawesi | 1700 | 1443 |
| 188 | Lamandau | Central Kalimantan | 1318 | 1442 |
| 189 | Rote Ndao | East Nusa Tenggara | 2130 | 1439 |
| 190 | Magelang | Central Java | 18557 | 1433 |
| 191 | Rejang Lebong | Bengkulu | 4011 | 1432 |
| 192 | Hulu Sungai Tengah | South Kalimantan | 3710 | 1431 |
| 193 | Bandung Barat | West Java | 23716 | 1422 |
| 194 | Agam | West Sumatra | 7544 | 1422 |
| 195 | Sikka | East Nusa Tenggara | 4561 | 1417 |
| 196 | Halmahera Utara | North Maluku | 2832 | 1416 |
| 197 | Ponorogo | East Java | 13614 | 1402 |
| 198 | Kotawaringin Timur | Central Kalimantan | 5800 | 1399 |
| 199 | Kaimana | West Papua | 891 | 1384 |
| 200 | Sorong | West Papua | 1697 | 1378 |
| 201 | Kota Sabang | Aceh | 581 | 1377 |
| 202 | Kepahiang | Bengkulu | 2087 | 1373 |
| 203 | Kota Kotamobagu | North Sulawesi | 1695 | 1373 |
| 204 | Gowa | South Sulawesi | 10428 | 1372 |
| 205 | Katingan | Central Kalimantan | 2162 | 1370 |
| 206 | Kota Bandar Lampung | Lampung | 16225 | 1368 |
| 207 | Kota Tidore Kepulauan | North Maluku | 1569 | 1367 |
| 208 | Nganjuk | East Java | 15302 | 1362 |
| 209 | Banjar | South Kalimantan | 7514 | 1360 |
| 210 | Cirebon | West Java | 29736 | 1358 |
| 211 | Madiun | East Java | 10131 | 1352 |
| 212 | Maros | South Sulawesi | 5219 | 1351 |
| 213 | Garut | West Java | 30831 | 1350 |
| 214 | Kota Ternate | North Maluku | 2956 | 1347 |
| 215 | Pangandaran | West Java | 5710 | 1344 |
| 216 | Tanah Datar | West Sumatra | 4947 | 1327 |
| 217 | Kuningan | West Java | 15471 | 1321 |
| 218 | Kapuas | Central Kalimantan | 5454 | 1312 |
| 219 | Kebumen | Central Java | 18118 | 1305 |
| 220 | Muaro Jambi | Jambi | 4771 | 1296 |
| 221 | Ciamis | West Java | 16159 | 1289 |
| 222 | Sabu Raijua | East Nusa Tenggara | 1189 | 1259 |
| 223 | Bandung | West Java | 44522 | 1250 |
| 224 | Bengkulu Utara | Bengkulu | 3574 | 1242 |
| 225 | Rembang | Central Java | 7938 | 1240 |
| 226 | Tegal | Central Java | 19400 | 1233 |
| 227 | Situbondo | East Java | 8470 | 1225 |
| 228 | Muko Muko | Bengkulu | 2210 | 1207 |
| 229 | Sumba Barat | East Nusa Tenggara | 1793 | 1204 |
| 230 | Kampar | Riau | 9084 | 1204 |
| 231 | Kepulauan Sangihe | North Sulawesi | 1712 | 1201 |
| 232 | Trenggalek | East Java | 8953 | 1193 |
| 233 | Dharmasraya | West Sumatra | 2561 | 1189 |
| 234 | Soppeng | South Sulawesi | 2803 | 1182 |
| 235 | Pringsewu | Lampung | 4738 | 1172 |
| 236 | Kediri | East Java | 19468 | 1171 |
| 237 | Blora | Central Java | 10563 | 1168 |
| 238 | Merauke | Papua | 2641 | 1162 |
| 239 | Mamuju | West Sulawesi | 3538 | 1161 |
| 240 | Wonogiri | Central Java | 12672 | 1161 |
| 241 | Jombang | East Java | 15419 | 1148 |
| 242 | Bengkayang | West Kalimantan | 3311 | 1147 |
| 243 | Pangkajene Kepulauan | South Sulawesi | 3999 | 1142 |
| 244 | Tabalong | South Kalimantan | 2774 | 1138 |
| 245 | Kota Gunungsitoli | North Sumatra | 1541 | 1133 |
| 246 | Deli Serdang | North Sumatra | 20810 | 1128 |
| 247 | Tolikara | Papua | 2439 | 1113 |
| 248 | Lampung Barat | Lampung | 3406 | 1113 |
| 249 | Batanghari | Jambi | 3389 | 1110 |
| 250 | Boyolali | Central Java | 11662 | 1106 |
| 251 | Solok Selatan | West Sumatra | 1993 | 1094 |
| 252 | Parigi Moutong | Central Sulawesi | 4908 | 1093 |
| 253 | Karangasem | Bali | 5646 | 1092 |
| 254 | Luwu Utara | South Sulawesi | 3559 | 1085 |
| 255 | Kolaka | Southeast Sulawesi | 2540 | 1083 |
| 256 | Barru | South Sulawesi | 1968 | 1079 |
| 257 | Purbalingga | Central Java | 10732 | 1070 |
| 258 | Barito Utara | Central Kalimantan | 1653 | 1058 |
| 259 | Indramayu | West Java | 19652 | 1056 |
| 260 | Banjarnegara | Central Java | 10688 | 1045 |
| 261 | Sorong Selatan | West Papua | 564 | 1044 |
| 262 | Ngawi | East Java | 9502 | 1040 |
| 263 | Bone Bolango | Gorontalo | 1718 | 1039 |
| 264 | Pulang Pisau | Central Kalimantan | 1370 | 1032 |
| 265 | Manggarai Timur | East Nusa Tenggara | 2780 | 1031 |
| 266 | Pasangkayu | West Sulawesi | 2201 | 1029 |
| 267 | Banyuwangi | East Java | 17908 | 1025 |
| 268 | Sijunjung | West Sumatra | 2438 | 1020 |
| 269 | Kepulauan Meranti | Riau | 2134 | 1019 |
| 270 | Mempawah | West Kalimantan | 3116 | 1017 |
| 271 | Hulu Sungai Selatan | South Kalimantan | 2349 | 1014 |
| 272 | Rokan Hulu | Riau | 5633 | 1010 |
| 273 | Kepulauan Selayar | South Sulawesi | 1406 | 1008 |
| 274 | Kotabaru | South Kalimantan | 3244 | 1001 |
| 275 | Lebak | Banten | 12924 | 999 |
| 276 | Minahasa Selatan | North Sulawesi | 2382 | 998 |
| 277 | Bondowoso | East Java | 7939 | 995 |
| 278 | Toba Samosir | North Sumatra | 2105 | 991 |
| 279 | Sumbawa Barat | West Nusa Tenggara | 1386 | 982 |
| 280 | Majalengka | West Java | 12722 | 973 |
| 281 | Banggai Kepulauan | Central Sulawesi | 1193 | 968 |
| 282 | Blitar | East Java | 12031 | 965 |
| 283 | Kota Tebing Tinggi | North Sumatra | 1680 | 964 |
| 284 | Pelalawan | Riau | 3565 | 960 |
| 285 | Nagekeo | East Nusa Tenggara | 1578 | 958 |
| 286 | Manokwari Selatan | West Papua | 350 | 951 |
| 287 | Jayawijaya | Papua | 2580 | 947 |
| 288 | Kota Lhokseumawe | Aceh | 1791 | 945 |
| 289 | Dompu | West Nusa Tenggara | 2116 | 945 |
| 290 | Sumedang | West Java | 10872 | 942 |
| 291 | Lumajang | East Java | 10598 | 939 |
| 292 | Demak | Central Java | 10871 | 938 |
| 293 | Takalar | South Sulawesi | 2755 | 938 |
| 294 | Kolaka Utara | Southeast Sulawesi | 1295 | 934 |
| 295 | Konawe Utara | Southeast Sulawesi | 646 | 933 |
| 296 | Indragiri Hilir | Riau | 5860 | 930 |
| 297 | Serang | Banten | 13780 | 929 |
| 298 | Kota Binjai | North Sumatra | 2642 | 918 |
| 299 | Bengkulu Tengah | Bengkulu | 1047 | 914 |
| 300 | Mojokerto | East Java | 10356 | 910 |
| 301 | Batang | Central Java | 7236 | 910 |
| 302 | Pati | Central Java | 12054 | 909 |
| 303 | Malang | East Java | 23120 | 907 |
| 304 | Keerom | Papua | 574 | 901 |
| 305 | Kota Sibolga | North Sumatra | 835 | 897 |
| 306 | Dairi | North Sumatra | 2823 | 894 |
| 307 | Kepulauan Tanimbar | Maluku | 1136 | 892 |
| 308 | Halmahera Timur | North Maluku | 837 | 876 |
| 309 | Landak | West Kalimantan | 3512 | 875 |
| 310 | Pemalang | Central Java | 13102 | 875 |
| 311 | Gorontalo | Gorontalo | 3489 | 873 |
| 312 | Tanjung Jabung Barat | Jambi | 2773 | 866 |
| 313 | Cilacap | Central Java | 16710 | 862 |
| 314 | Sintang | West Kalimantan | 3536 | 861 |
| 315 | Kepulauan Talaud | North Sulawesi | 833 | 858 |
| 316 | Kupang | East Nusa Tenggara | 3254 | 854 |
| 317 | Rokan Hilir | Riau | 5425 | 847 |
| 318 | Solok | West Sumatra | 3284 | 847 |
| 319 | Tulungagung | East Java | 9463 | 846 |
| 320 | Mamuju Tengah | West Sulawesi | 1201 | 838 |
| 321 | Belu | East Nusa Tenggara | 1892 | 837 |
| 322 | Mamasa | West Sulawesi | 1657 | 828 |
| 323 | Supiori | Papua | 198 | 820 |
| 324 | Seruyan | Central Kalimantan | 1209 | 816 |
| 325 | Muara Enim | South Sumatra | 4725 | 814 |
| 326 | Pesisir Barat | Lampung | 1309 | 814 |
| 327 | Melawi | West Kalimantan | 1892 | 810 |
| 328 | Lampung Utara | Lampung | 5031 | 809 |
| 329 | Subang | West Java | 12646 | 804 |
| 330 | Banggai Laut | Central Sulawesi | 544 | 792 |
| 331 | Lima Puluh Kota | West Sumatra | 3021 | 789 |
| 332 | Pekalongan | Central Java | 7501 | 785 |
| 333 | Jember | East Java | 20134 | 784 |
| 334 | Sumbawa | West Nusa Tenggara | 4020 | 778 |
| 335 | Konawe | Southeast Sulawesi | 2004 | 774 |
| 336 | Kubu Raya | West Kalimantan | 4655 | 766 |
| 337 | Bolaang Mongondow Utara | North Sulawesi | 644 | 759 |
| 338 | Pesisir Selatan | West Sumatra | 3877 | 758 |
| 339 | Nabire | Papua | 1299 | 756 |
| 340 | Pandeglang | Banten | 9119 | 754 |
| 341 | Polewali Mandar | West Sulawesi | 3874 | 739 |
| 342 | Grobogan | Central Java | 10872 | 738 |
| 343 | Padang Pariaman | West Sumatra | 3177 | 737 |
| 344 | Kayong Utara | West Kalimantan | 932 | 732 |
| 345 | Gorontalo Utara | Gorontalo | 917 | 729 |
| 346 | Kepulauan Siau Tagulandang Biaro | North Sulawesi | 538 | 728 |
| 347 | Donggala | Central Sulawesi | 2188 | 722 |
| 348 | Majene | West Sulawesi | 1252 | 719 |
| 349 | Tapanuli Utara | North Sumatra | 2271 | 715 |
| 350 | Probolinggo | East Java | 8071 | 709 |
| 351 | Tuban | East Java | 8527 | 704 |
| 352 | Musi Rawas | South Sumatra | 2834 | 701 |
| 353 | Kolaka Timur | Southeast Sulawesi | 871 | 701 |
| 354 | Pahuwato | Gorontalo | 1028 | 700 |
| 355 | Pesawaran | Lampung | 3306 | 699 |
| 356 | Bantaeng | South Sulawesi | 1400 | 696 |
| 357 | Pasuruan | East Java | 11063 | 695 |
| 358 | Gayo Lues | Aceh | 699 | 692 |
| 359 | Bangkalan | East Java | 7313 | 679 |
| 360 | Tebo | Jambi | 2216 | 676 |
| 361 | Bungo | Jambi | 2301 | 671 |
| 362 | Halmahera Tengah | North Maluku | 367 | 665 |
| 363 | Toraja Utara | South Sulawesi | 1640 | 664 |
| 364 | Aceh Timur | Aceh | 1411 | 657 |
| 365 | Sekadau | West Kalimantan | 1406 | 656 |
| 366 | Lombok Barat | West Nusa Tenggara | 4712 | 654 |
| 367 | Bolaang Mongondow Timur | North Sulawesi | 578 | 653 |
| 368 | Bojonegoro | East Java | 8716 | 652 |
| 369 | Tanjung Jabung Timur | Jambi | 1481 | 650 |
| 370 | Lahat | South Sumatra | 2818 | 648 |
| 371 | Sumba Barat Daya | East Nusa Tenggara | 2023 | 646 |
| 372 | Lebong | Bengkulu | 692 | 645 |
| 373 | Pakpak Bharat | North Sumatra | 349 | 643 |
| 374 | Aceh Tamiang | Aceh | 1901 | 639 |
| 375 | Flores Timur | East Nusa Tenggara | 1815 | 639 |
| 376 | Halmahera Selatan | North Maluku | 1576 | 626 |
| 377 | Kota Langsa | Aceh | 1168 | 623 |
| 378 | Aceh Barat | Aceh | 1219 | 622 |
| 379 | Brebes | Central Java | 11964 | 621 |
| 380 | Lamongan | East Java | 8522 | 621 |
| 381 | Lampung Selatan | Lampung | 6463 | 618 |
| 382 | Jeneponto | South Sulawesi | 2545 | 615 |
| 383 | Aceh Singkil | Aceh | 778 | 613 |
| 384 | Boalemo | Gorontalo | 903 | 611 |
| 385 | Kota Pagar Alam | South Sumatra | 891 | 610 |
| 386 | Kota Padang Sidempuan | North Sumatra | 1374 | 605 |
| 387 | Pasaman Barat | West Sumatra | 2617 | 603 |
| 388 | Cianjur | West Java | 13673 | 596 |
| 389 | Pidie | Aceh | 2639 | 596 |
| 390 | Lampung Timur | Lampung | 6708 | 596 |
| 391 | Alor | East Nusa Tenggara | 1275 | 586 |
| 392 | Karo | North Sumatra | 2387 | 586 |
| 393 | Sanggau | West Kalimantan | 2851 | 585 |
| 394 | Bolaang Mongondow Selatan | North Sulawesi | 415 | 580 |
| 395 | Sukabumi | West Java | 14700 | 572 |
| 396 | Aceh Jaya | Aceh | 525 | 570 |
| 397 | Ketapang | West Kalimantan | 3212 | 560 |
| 398 | Kota Tual | Maluku | 496 | 560 |
| 399 | Sidenreng Rappang | South Sulawesi | 1760 | 551 |
| 400 | Tulang Bawang Barat | Lampung | 1515 | 548 |
| 401 | Bengkulu Selatan | Bengkulu | 927 | 547 |
| 402 | Sambas | West Kalimantan | 3473 | 545 |
| 403 | Sumenep | East Java | 6183 | 545 |
| 404 | Musi Banyuasin | South Sumatra | 3190 | 542 |
| 405 | Penukal Abab Lematang Ilir | South Sumatra | 977 | 536 |
| 406 | Batu Bara | North Sumatra | 1963 | 529 |
| 407 | Bombana | Southeast Sulawesi | 751 | 525 |
| 408 | Tasikmalaya | West Java | 9355 | 521 |
| 409 | Musi Rawas Utara | South Sumatra | 1002 | 517 |
| 410 | Pasaman | West Sumatra | 1606 | 516 |
| 411 | Bulukumba | South Sulawesi | 2245 | 511 |
| 412 | Merangin | Jambi | 1766 | 504 |
| 413 | Pulau Taliabu | North Maluku | 297 | 501 |
| 414 | Tanggamus | Lampung | 3224 | 497 |
| 415 | Pidie Jaya | Aceh | 798 | 494 |
| 416 | Maluku Barat Daya | Maluku | 363 | 491 |
| 417 | Konawe Selatan | Southeast Sulawesi | 1515 | 485 |
| 418 | Halmahera Barat | North Maluku | 658 | 485 |
| 419 | Timor Tengah Selatan | East Nusa Tenggara | 2239 | 478 |
| 420 | Ogan Komering Ulu Timur | South Sumatra | 3057 | 475 |
| 421 | Sarmi | Papua | 194 | 471 |
| 422 | Banyuasin | South Sumatra | 3790 | 465 |
| 423 | Sarolangun | Jambi | 1287 | 461 |
| 424 | Malaka | East Nusa Tenggara | 884 | 455 |
| 425 | Simeulue | Aceh | 426 | 455 |
| 426 | Kepulauan Sula | North Maluku | 477 | 452 |
| 427 | Pinrang | South Sulawesi | 1811 | 446 |
| 428 | Wajo | South Sulawesi | 1670 | 445 |
| 429 | Timor Tengah Utara | East Nusa Tenggara | 1158 | 434 |
| 430 | Wakatobi | Southeast Sulawesi | 490 | 427 |
| 431 | Serdang Bedagai | North Sumatra | 2802 | 425 |
| 432 | Simalungun | North Sumatra | 4377 | 423 |
| 433 | Konawe Kepulauan | Southeast Sulawesi | 159 | 422 |
| 434 | Luwu | South Sulawesi | 1559 | 421 |
| 435 | Labuhanbatu | North Sumatra | 2090 | 415 |
| 436 | Kaur | Bengkulu | 535 | 409 |
| 437 | Kota Tanjung Balai | North Sumatra | 718 | 406 |
| 438 | Bireuen | Aceh | 1781 | 404 |
| 439 | Aceh Selatan | Aceh | 942 | 399 |
| 440 | Bolaang Mongondow | North Sulawesi | 987 | 394 |
| 441 | Sampang | East Java | 3384 | 393 |
| 442 | Lampung Tengah | Lampung | 5703 | 385 |
| 443 | Mesuji | Lampung | 885 | 384 |
| 444 | Ogan Ilir | South Sumatra | 1562 | 377 |
| 445 | Enrekang | South Sulawesi | 850 | 375 |
| 446 | Buton Utara | Southeast Sulawesi | 247 | 372 |
| 447 | Lombok Tengah | West Nusa Tenggara | 3883 | 371 |
| 448 | Buton | Southeast Sulawesi | 437 | 368 |
| 449 | Kota Subulussalam | Aceh | 321 | 368 |
| 450 | Bima | West Nusa Tenggara | 1944 | 366 |
| 451 | Pamekasan | East Java | 2978 | 360 |
| 452 | Kapuas Hulu | West Kalimantan | 885 | 359 |
| 453 | Bone | South Sulawesi | 2880 | 356 |
| 454 | Buton Tengah | Southeast Sulawesi | 416 | 352 |
| 455 | Seluma | Bengkulu | 747 | 351 |
| 456 | Asahan | North Sumatra | 2757 | 350 |
| 457 | Maluku Tengah | Maluku | 1484 | 348 |
| 458 | Nagan Raya | Aceh | 584 | 340 |
| 459 | Humbang Hasundutan | North Sumatra | 681 | 340 |
| 460 | Muna | Southeast Sulawesi | 749 | 336 |
| 461 | Bener Meriah | Aceh | 527 | 328 |
| 462 | Tulang Bawang | Lampung | 1364 | 320 |
| 463 | Ogan Komering Ulu | South Sumatra | 1170 | 319 |
| 464 | Kepulauan Yapen | Papua | 359 | 318 |
| 465 | Way Kanan | Lampung | 1502 | 314 |
| 466 | Tapanuli Tengah | North Sumatra | 1119 | 308 |
| 467 | Labuhanbatu Utara | North Sumatra | 1199 | 304 |
| 468 | Labuhanbatu Selatan | North Sumatra | 962 | 298 |
| 469 | Lombok Utara | West Nusa Tenggara | 714 | 296 |
| 470 | Buru | Maluku | 394 | 289 |
| 471 | Maluku Tenggara | Maluku | 369 | 289 |
| 472 | Tapanuli Selatan | North Sumatra | 890 | 286 |
| 473 | Asmat | Papua | 306 | 285 |
| 474 | Tambrauw | West Papua | 89 | 278 |
| 475 | Lombok Timur | West Nusa Tenggara | 3587 | 276 |
| 476 | Aceh Barat Daya | Aceh | 414 | 272 |
| 477 | Langkat | North Sumatra | 2734 | 261 |
| 478 | Nias | North Sumatra | 393 | 255 |
| 479 | Kerinci | Jambi | 601 | 248 |
| 480 | Muna Barat | Southeast Sulawesi | 190 | 227 |
| 481 | Nias Barat | North Sumatra | 201 | 209 |
| 482 | Aceh Utara | Aceh | 1115 | 190 |
| 483 | Ogan Komering Ulu Selatan | South Sumatra | 787 | 189 |
| 484 | Mappi | Papua | 199 | 185 |
| 485 | Buru Selatan | Maluku | 139 | 182 |
| 486 | Buton Selatan | Southeast Sulawesi | 174 | 177 |
| 487 | Aceh Tengah | Aceh | 386 | 171 |
| 488 | Empat Lawang | South Sumatra | 565 | 169 |
| 489 | Padang Lawas | North Sumatra | 417 | 159 |
| 490 | Nias Utara | North Sumatra | 239 | 158 |
| 491 | Seram Bagian Barat | Maluku | 325 | 154 |
| 492 | Mandailing Natal | North Sumatra | 739 | 152 |
| 493 | Waropen | Papua | 50 | 139 |
| 494 | Ogan Komering Ilir | South Sumatra | 983 | 135 |
| 495 | Mamberamo Tengah | Papua | 63 | 131 |
| 496 | Aceh Tenggara | Aceh | 550 | 130 |
| 497 | Padang Lawas Utara | North Sumatra | 307 | 114 |
| 498 | Seram Bagian Timur | Maluku | 136 | 100 |
| 499 | Maybrat | West Papua | 44 | 100 |
| 500 | Yalimo | Papua | 100 | 97 |
| 501 | Nias Selatan | North Sumatra | 295 | 81 |
| 502 | Pegunungan Bintang | Papua | 88 | 79 |
| 503 | Lanny Jaya | Papua | 142 | 70 |
| 504 | Mamberamo Raya | Papua | 25 | 66 |
| 505 | Toli Toli | Central Sulawesi | 148 | 59 |
| 506 | Yahukimo | Papua | 184 | 52 |
| 507 | Puncak Jaya | Papua | 77 | 35 |
| 508 | Paniai | Papua | 40 | 33 |
| 509 | Nduga | Papua | 31 | 28 |
| 510 | Pegunungan Arfak | West Papua | 8 | 21 |
| 511 | Puncak | Papua | 36 | 20 |
| 512 | Dogiyai | Papua | 12 | 10 |
| 513 | Deiyai | Papua | 8 | 9 |
| 514 | Intan Jaya | Papua | 11 | 8 |

**Supplementary Table 2. List of districts ranked based on their cumulative COVID-19 mortality rate in Indonesia**

| **Rank** | **District** | **Province** | **Number of deaths** | **Mortality per 100,000 population** |
| --- | --- | --- | --- | --- |
| 1 | Kota Balikpapan | East Kalimantan | 1903 | 284 |
| 2 | Kota Semarang | Central Java | 4395 | 262 |
| 3 | Kota Madiun | East Java | 526 | 254 |
| 4 | Kota Magelang | Central Java | 325 | 250 |
| 5 | Kota Yogyakarta | Yogyakarta | 1023 | 247 |
| 6 | Kota Bontang | East Kalimantan | 362 | 199 |
| 7 | Kota Surakarta | Central Java | 1118 | 194 |
| 8 | Kota Palangkaraya | Central Kalimantan | 491 | 184 |
| 9 | Kota Tanjung Pinang | Riau Islands | 405 | 183 |
| 10 | Berau | East Kalimantan | 414 | 178 |
| 11 | Kutai Barat | East Kalimantan | 280 | 171 |
| 12 | Kota Blitar | East Java | 265 | 168 |
| 13 | Kota Mojokerto | East Java | 233 | 168 |
| 14 | Sleman | Yogyakarta | 1791 | 167 |
| 15 | Kota Denpasar | Bali | 1061 | 163 |
| 16 | Kota Jakarta Pusat | Jakarta | 1801 | 157 |
| 17 | Kota Banjarbaru | South Kalimantan | 372 | 157 |
| 18 | Kota Cirebon | West Java | 530 | 156 |
| 19 | Kota Tarakan | North Kalimantan | 352 | 152 |
| 20 | Kota Probolinggo | East Java | 366 | 152 |
| 21 | Bulungan | North Kalimantan | 207 | 147 |
| 22 | Bantul | Yogyakarta | 1390 | 146 |
| 23 | Badung | Bali | 698 | 142 |
| 24 | Kota Banda Aceh | Aceh | 348 | 142 |
| 25 | Magetan | East Java | 987 | 141 |
| 26 | Ponorogo | East Java | 1362 | 140 |
| 27 | Trenggalek | East Java | 1049 | 140 |
| 28 | Kota Pangkal Pinang | Bangka Belitung Islands | 299 | 139 |
| 29 | Kota Tegal | Central Java | 393 | 137 |
| 30 | Kudus | Central Java | 1180 | 137 |
| 31 | Belitung | Bangka Belitung Islands | 237 | 136 |
| 32 | Kota Pekanbaru | Riau | 1306 | 136 |
| 33 | Penajam Paser Utara | East Kalimantan | 232 | 134 |
| 34 | Kota Kediri | East Java | 391 | 133 |
| 35 | Blitar | East Java | 1635 | 131 |
| 36 | Kota Malang | East Java | 1124 | 131 |
| 37 | Kota Batu | East Java | 272 | 128 |
| 38 | Tabanan | Bali | 582 | 128 |
| 39 | Situbondo | East Java | 884 | 128 |
| 40 | Sukoharjo | Central Java | 1139 | 126 |
| 41 | Kota Tomohon | North Sulawesi | 124 | 124 |
| 42 | Kutai Kartanegara | East Kalimantan | 847 | 122 |
| 43 | Kota Pasuruan | East Java | 254 | 120 |
| 44 | Kota Jakarta Selatan | Jakarta | 2779 | 119 |
| 45 | Kota Depok | West Java | 2196 | 118 |
| 46 | Kota Jakarta Timur | Jakarta | 3746 | 118 |
| 47 | Jombang | East Java | 1578 | 117 |
| 48 | Kota Jakarta Barat | Jakarta | 2980 | 117 |
| 49 | Bangka | Bangka Belitung Islands | 372 | 117 |
| 50 | Tana Tidung | North Kalimantan | 28 | 116 |
| 51 | Bintan | Riau Islands | 181 | 116 |
| 52 | Wonogiri | Central Java | 1261 | 116 |
| 53 | Klaten | Central Java | 1509 | 114 |
| 54 | Malinau | North Kalimantan | 91 | 113 |
| 55 | Sragen | Central Java | 1117 | 112 |
| 56 | Kota Sabang | Aceh | 47 | 111 |
| 57 | Kota Metro | Lampung | 187 | 109 |
| 58 | Kutai Timur | East Kalimantan | 451 | 107 |
| 59 | Rembang | Central Java | 677 | 106 |
| 60 | Bangli | Bali | 279 | 104 |
| 61 | Karanganyar | Central Java | 962 | 104 |
| 62 | Paser | East Kalimantan | 274 | 103 |
| 63 | Gunungkidul | Yogyakarta | 792 | 103 |
| 64 | Kota Pekalongan | Central Java | 320 | 102 |
| 65 | Kota Jakarta Utara | Jakarta | 1840 | 101 |
| 66 | Kepulauan Anambas | Riau Islands | 47 | 100 |
| 67 | Banyuwangi | East Java | 1744 | 100 |
| 68 | Bangka Tengah | Bangka Belitung Islands | 185 | 99 |
| 69 | Purworejo | Central Java | 777 | 98 |
| 70 | Kota Prabumulih | South Sumatra | 189 | 96 |
| 71 | Ngawi | East Java | 875 | 96 |
| 72 | Bondowoso | East Java | 760 | 95 |
| 73 | Kota Salatiga | Central Java | 183 | 94 |
| 74 | Madiun | East Java | 694 | 93 |
| 75 | Mahakam Ulu | East Kalimantan | 28 | 92 |
| 76 | Kota Padang Panjang | West Sumatra | 53 | 91 |
| 77 | Kota Samarinda | East Kalimantan | 722 | 91 |
| 78 | Kota Surabaya | East Java | 2649 | 90 |
| 79 | Tanah Bumbu | South Kalimantan | 285 | 89 |
| 80 | Lumajang | East Java | 978 | 87 |
| 81 | Banggai | Central Sulawesi | 318 | 86 |
| 82 | Kota Banjarmasin | South Kalimantan | 577 | 86 |
| 83 | Bangka Selatan | Bangka Belitung Islands | 156 | 86 |
| 84 | Klungkung | Bali | 184 | 86 |
| 85 | Pringsewu | Lampung | 343 | 85 |
| 86 | Kota Dumai | Riau | 255 | 84 |
| 87 | Lingga | Riau Islands | 85 | 84 |
| 88 | Kulon Progo | Yogyakarta | 374 | 84 |
| 89 | Kota Sukabumi | West Java | 290 | 83 |
| 90 | Karawang | West Java | 1925 | 83 |
| 91 | Kebumen | Central Java | 1149 | 83 |
| 92 | Kotawaringin Barat | Central Kalimantan | 210 | 83 |
| 93 | Kota Bukittinggi | West Sumatra | 101 | 82 |
| 94 | Semarang | Central Java | 834 | 81 |
| 95 | Kota Gorontalo | Gorontalo | 160 | 80 |
| 96 | Kota Solok | West Sumatra | 59 | 79 |
| 97 | Sukamara | Central Kalimantan | 49 | 79 |
| 98 | Kota Pontianak | West Kalimantan | 527 | 79 |
| 99 | Pati | Central Java | 1044 | 79 |
| 100 | Aceh Besar | Aceh | 307 | 78 |
| 101 | Kota Cilegon | Banten | 328 | 77 |
| 102 | Kota Batam | Riau Islands | 854 | 77 |
| 103 | Tuban | East Java | 929 | 77 |
| 104 | Kota Palembang | South Sumatra | 1238 | 76 |
| 105 | Kota Kupang | East Nusa Tenggara | 336 | 76 |
| 106 | Balangan | South Kalimantan | 100 | 76 |
| 107 | Poso | Central Sulawesi | 188 | 76 |
| 108 | Kediri | East Java | 1255 | 75 |
| 109 | Kota Tasikmalaya | West Java | 542 | 75 |
| 110 | Bengkalis | Riau | 415 | 75 |
| 111 | Nunukan | North Kalimantan | 135 | 74 |
| 112 | Belitung Timur | Bangka Belitung Islands | 93 | 74 |
| 113 | Jembrana | Bali | 240 | 73 |
| 114 | Kendal | Central Java | 738 | 73 |
| 115 | Bangka Barat | Bangka Belitung Islands | 145 | 73 |
| 116 | Morowali Utara | Central Sulawesi | 88 | 72 |
| 117 | Siak | Riau | 306 | 71 |
| 118 | Kota Makassar | South Sulawesi | 1049 | 71 |
| 119 | Karangasem | Bali | 366 | 71 |
| 120 | Buleleng | Bali | 582 | 71 |
| 121 | Nganjuk | East Java | 793 | 71 |
| 122 | Gianyar | Bali | 353 | 70 |
| 123 | Demak | Central Java | 814 | 70 |
| 124 | Tolikara | Papua | 152 | 69 |
| 125 | Tanah Laut | South Kalimantan | 241 | 69 |
| 126 | Bangkalan | East Java | 735 | 68 |
| 127 | Kota Bandar Lampung | Lampung | 801 | 68 |
| 128 | Blora | Central Java | 607 | 67 |
| 129 | Kota Banjar | West Java | 133 | 65 |
| 130 | Pidie | Aceh | 283 | 64 |
| 131 | Rokan Hulu | Riau | 355 | 64 |
| 132 | Tojo Una Una | Central Sulawesi | 104 | 63 |
| 133 | Kota Mataram | West Nusa Tenggara | 270 | 63 |
| 134 | Karimun | Riau Islands | 157 | 63 |
| 135 | Hulu Sungai Tengah | South Kalimantan | 162 | 62 |
| 136 | Merauke | Papua | 140 | 62 |
| 137 | Kota Padang | West Sumatra | 559 | 62 |
| 138 | Kota Sawahlunto | West Sumatra | 41 | 61 |
| 139 | Purwakarta | West Java | 582 | 61 |
| 140 | Kota Palu | Central Sulawesi | 227 | 61 |
| 141 | Wonosobo | Central Java | 541 | 61 |
| 142 | Temanggung | Central Java | 480 | 61 |
| 143 | Banyumas | Central Java | 1073 | 60 |
| 144 | Kota Pare Pare | South Sulawesi | 88 | 59 |
| 145 | Gresik | East Java | 743 | 58 |
| 146 | Tegal | Central Java | 913 | 58 |
| 147 | Jember | East Java | 1469 | 57 |
| 148 | Kota Manado | North Sulawesi | 270 | 57 |
| 149 | Jepara | Central Java | 675 | 57 |
| 150 | Minahasa Tenggara | North Sulawesi | 66 | 56 |
| 151 | Minahasa | North Sulawesi | 190 | 56 |
| 152 | Kepulauan Siau Tagulandang Biaro | North Sulawesi | 41 | 56 |
| 153 | Lampung Timur | Lampung | 617 | 55 |
| 154 | Kampar | Riau | 411 | 54 |
| 155 | Tapin | South Kalimantan | 101 | 54 |
| 156 | Hulu Sungai Utara | South Kalimantan | 123 | 53 |
| 157 | Pacitan | East Java | 315 | 53 |
| 158 | Garut | West Java | 1197 | 52 |
| 159 | Kota Tangerang Selatan | Banten | 668 | 52 |
| 160 | Cilacap | Central Java | 1009 | 52 |
| 161 | Sorong Selatan | West Papua | 28 | 52 |
| 162 | Magelang | Central Java | 669 | 52 |
| 163 | Tulang Bawang Barat | Lampung | 142 | 51 |
| 164 | Natuna | Riau Islands | 42 | 51 |
| 165 | Sumba Timur | East Nusa Tenggara | 124 | 51 |
| 166 | Sidoarjo | East Java | 980 | 51 |
| 167 | Kota Bogor | West Java | 533 | 51 |
| 168 | Pulang Pisau | Central Kalimantan | 67 | 50 |
| 169 | Batang | Central Java | 399 | 50 |
| 170 | Pemalang | Central Java | 746 | 50 |
| 171 | Pesawaran | Lampung | 235 | 50 |
| 172 | Kuantan Singingi | Riau | 164 | 49 |
| 173 | Kota Langsa | Aceh | 92 | 49 |
| 174 | Pelalawan | Riau | 182 | 49 |
| 175 | Aceh Tamiang | Aceh | 144 | 48 |
| 176 | Morowali | Central Sulawesi | 71 | 48 |
| 177 | Indragiri Hulu | Riau | 207 | 48 |
| 178 | Kota Tidore Kepulauan | North Maluku | 54 | 47 |
| 179 | Bojonegoro | East Java | 627 | 47 |
| 180 | Banjarnegara | Central Java | 479 | 47 |
| 181 | Kota Ambon | Maluku | 160 | 46 |
| 182 | Kota Jayapura | Papua | 193 | 46 |
| 183 | Tanah Datar | West Sumatra | 169 | 45 |
| 184 | Brebes | Central Java | 871 | 45 |
| 185 | Teluk Wondama | West Papua | 20 | 45 |
| 186 | Sigi | Central Sulawesi | 114 | 45 |
| 187 | Probolinggo | East Java | 504 | 44 |
| 188 | Kotabaru | South Kalimantan | 143 | 44 |
| 189 | Kota Palopo | South Sulawesi | 80 | 44 |
| 190 | Sintang | West Kalimantan | 180 | 44 |
| 191 | Kota Lhokseumawe | Aceh | 83 | 44 |
| 192 | Lamandau | Central Kalimantan | 40 | 44 |
| 193 | Teluk Bintuni | West Papua | 35 | 44 |
| 194 | Penukal Abab Lematang Ilir | South Sumatra | 79 | 43 |
| 195 | Fak Fak | West Papua | 38 | 43 |
| 196 | Lampung Barat | Lampung | 131 | 43 |
| 197 | Bengkulu Utara | Bengkulu | 123 | 43 |
| 198 | Pasuruan | East Java | 678 | 43 |
| 199 | Buol | Central Sulawesi | 60 | 42 |
| 200 | Ogan Komering Ulu Timur | South Sumatra | 272 | 42 |
| 201 | Banggai Laut | Central Sulawesi | 29 | 42 |
| 202 | Muara Enim | South Sumatra | 245 | 42 |
| 203 | Kota Jambi | Jambi | 260 | 42 |
| 204 | Kota Bekasi | West Java | 1025 | 42 |
| 205 | Barito Selatan | Central Kalimantan | 54 | 42 |
| 206 | Kota Pariaman | West Sumatra | 39 | 42 |
| 207 | Cirebon | West Java | 908 | 41 |
| 208 | Minahasa Utara | North Sulawesi | 92 | 41 |
| 209 | Pekalongan | Central Java | 396 | 41 |
| 210 | Kota Lubuk Linggau | South Sumatra | 93 | 41 |
| 211 | Purbalingga | Central Java | 408 | 41 |
| 212 | Lampung Tengah | Lampung | 600 | 41 |
| 213 | Lembata | East Nusa Tenggara | 56 | 40 |
| 214 | Kota Bima | West Nusa Tenggara | 60 | 40 |
| 215 | Agam | West Sumatra | 212 | 40 |
| 216 | Boyolali | Central Java | 419 | 40 |
| 217 | Manokwari | West Papua | 76 | 40 |
| 218 | Kapuas | Central Kalimantan | 165 | 40 |
| 219 | Kota Medan | North Sumatra | 982 | 39 |
| 220 | Indramayu | West Java | 720 | 39 |
| 221 | Kotawaringin Timur | Central Kalimantan | 160 | 39 |
| 222 | Malang | East Java | 978 | 38 |
| 223 | Kota Payakumbuh | West Sumatra | 53 | 38 |
| 224 | Jayapura | Papua | 65 | 38 |
| 225 | Kota Kotamobagu | North Sulawesi | 47 | 38 |
| 226 | Batanghari | Jambi | 116 | 38 |
| 227 | Mamuju Tengah | West Sulawesi | 54 | 38 |
| 228 | Gorontalo | Gorontalo | 149 | 37 |
| 229 | Pasaman Barat | West Sumatra | 159 | 37 |
| 230 | Lampung Utara | Lampung | 227 | 37 |
| 231 | Kota Bengkulu | Bengkulu | 135 | 36 |
| 232 | Grobogan | Central Java | 536 | 36 |
| 233 | Nagan Raya | Aceh | 62 | 36 |
| 234 | Parigi Moutong | Central Sulawesi | 161 | 36 |
| 235 | Muko Muko | Bengkulu | 65 | 36 |
| 236 | Kepulauan Meranti | Riau | 73 | 35 |
| 237 | Katingan | Central Kalimantan | 55 | 35 |
| 238 | Ende | East Nusa Tenggara | 96 | 35 |
| 239 | Pangandaran | West Java | 148 | 35 |
| 240 | Manggarai | East Nusa Tenggara | 112 | 35 |
| 241 | Kota Sorong | West Papua | 96 | 34 |
| 242 | Aceh Timur | Aceh | 73 | 34 |
| 243 | Lima Puluh Kota | West Sumatra | 129 | 34 |
| 244 | Aceh Barat | Aceh | 66 | 34 |
| 245 | Rokan Hilir | Riau | 215 | 34 |
| 246 | Aceh Selatan | Aceh | 79 | 33 |
| 247 | Kota Bau Bau | Southeast Sulawesi | 52 | 33 |
| 248 | Hulu Sungai Selatan | South Kalimantan | 76 | 33 |
| 249 | Kota Ternate | North Maluku | 72 | 33 |
| 250 | Kota Cimahi | West Java | 181 | 33 |
| 251 | Kepulauan Selayar | South Sulawesi | 45 | 32 |
| 252 | Lamongan | East Java | 440 | 32 |
| 253 | Kota Pematangsiantar | North Sumatra | 87 | 32 |
| 254 | Manggarai Barat | East Nusa Tenggara | 84 | 32 |
| 255 | Sumba Tengah | East Nusa Tenggara | 28 | 32 |
| 256 | Aceh Jaya | Aceh | 29 | 31 |
| 257 | Kota Pagar Alam | South Sumatra | 46 | 31 |
| 258 | Bone Bolango | Gorontalo | 52 | 31 |
| 259 | Banjar | South Kalimantan | 173 | 31 |
| 260 | Kota Sibolga | North Sumatra | 29 | 31 |
| 261 | Kota Tangerang | Banten | 549 | 31 |
| 262 | Gunung Mas | Central Kalimantan | 42 | 31 |
| 263 | Solok | West Sumatra | 120 | 31 |
| 264 | Banggai Kepulauan | Central Sulawesi | 38 | 31 |
| 265 | Lahat | South Sumatra | 134 | 31 |
| 266 | Padang Pariaman | West Sumatra | 132 | 31 |
| 267 | Indragiri Hilir | Riau | 191 | 30 |
| 268 | Pangkajene Kepulauan | South Sulawesi | 106 | 30 |
| 269 | Kepulauan Sangihe | North Sulawesi | 43 | 30 |
| 270 | Kota Bitung | North Sulawesi | 68 | 30 |
| 271 | Ogan Komering Ulu | South Sumatra | 110 | 30 |
| 272 | Dharmasraya | West Sumatra | 64 | 30 |
| 273 | Barito Utara | Central Kalimantan | 46 | 29 |
| 274 | Polewali Mandar | West Sulawesi | 153 | 29 |
| 275 | Dairi | North Sumatra | 92 | 29 |
| 276 | Konawe | Southeast Sulawesi | 75 | 29 |
| 277 | Gayo Lues | Aceh | 29 | 29 |
| 278 | Sumbawa | West Nusa Tenggara | 147 | 28 |
| 279 | Sijunjung | West Sumatra | 68 | 28 |
| 280 | Sorong | West Papua | 35 | 28 |
| 281 | Musi Rawas Utara | South Sumatra | 55 | 28 |
| 282 | Soppeng | South Sulawesi | 67 | 28 |
| 283 | Kolaka Timur | Southeast Sulawesi | 35 | 28 |
| 284 | Kota Kendari | Southeast Sulawesi | 96 | 28 |
| 285 | Barito Timur | Central Kalimantan | 32 | 28 |
| 286 | Pesisir Barat | Lampung | 45 | 28 |
| 287 | Luwu Utara | South Sulawesi | 90 | 27 |
| 288 | Samosir | North Sumatra | 38 | 27 |
| 289 | Buton Utara | Southeast Sulawesi | 18 | 27 |
| 290 | Merangin | Jambi | 94 | 27 |
| 291 | Aceh Singkil | Aceh | 34 | 27 |
| 292 | Tulungagung | East Java | 285 | 25 |
| 293 | Bengkulu Tengah | Bengkulu | 29 | 25 |
| 294 | Boven Digoel | Papua | 16 | 25 |
| 295 | Lampung Selatan | Lampung | 264 | 25 |
| 296 | Ciamis | West Java | 315 | 25 |
| 297 | Boalemo | Gorontalo | 37 | 25 |
| 298 | Halmahera Utara | North Maluku | 50 | 25 |
| 299 | Rote Ndao | East Nusa Tenggara | 37 | 25 |
| 300 | Mesuji | Lampung | 57 | 25 |
| 301 | Solok Selatan | West Sumatra | 45 | 25 |
| 302 | Gorontalo Utara | Gorontalo | 31 | 25 |
| 303 | Bireuen | Aceh | 108 | 25 |
| 304 | Pamekasan | East Java | 202 | 24 |
| 305 | Sumenep | East Java | 275 | 24 |
| 306 | Pinrang | South Sulawesi | 98 | 24 |
| 307 | Kota Binjai | North Sumatra | 69 | 24 |
| 308 | Musi Banyuasin | South Sumatra | 141 | 24 |
| 309 | Kolaka Utara | Southeast Sulawesi | 33 | 24 |
| 310 | Bolaang Mongondow Timur | North Sulawesi | 21 | 24 |
| 311 | Wakatobi | Southeast Sulawesi | 27 | 24 |
| 312 | Majene | West Sulawesi | 41 | 24 |
| 313 | Minahasa Selatan | North Sulawesi | 56 | 23 |
| 314 | Donggala | Central Sulawesi | 71 | 23 |
| 315 | Deli Serdang | North Sumatra | 420 | 23 |
| 316 | Pesisir Selatan | West Sumatra | 115 | 22 |
| 317 | Pahuwato | Gorontalo | 33 | 22 |
| 318 | Asahan | North Sumatra | 176 | 22 |
| 319 | Sabu Raijua | East Nusa Tenggara | 21 | 22 |
| 320 | Kuningan | West Java | 260 | 22 |
| 321 | Pasaman | West Sumatra | 69 | 22 |
| 322 | Halmahera Barat | North Maluku | 30 | 22 |
| 323 | Kota Serang | Banten | 144 | 22 |
| 324 | Musi Rawas | South Sumatra | 89 | 22 |
| 325 | Buton | Southeast Sulawesi | 26 | 22 |
| 326 | Pandeglang | Banten | 265 | 22 |
| 327 | Kota Subulussalam | Aceh | 19 | 22 |
| 328 | Kaimana | West Papua | 14 | 22 |
| 329 | Konawe Utara | Southeast Sulawesi | 15 | 22 |
| 330 | Tanggamus | Lampung | 140 | 22 |
| 331 | Tanjung Jabung Barat | Jambi | 69 | 22 |
| 332 | Ngada | East Nusa Tenggara | 36 | 21 |
| 333 | Pidie Jaya | Aceh | 34 | 21 |
| 334 | Pasangkayu | West Sulawesi | 45 | 21 |
| 335 | Tebo | Jambi | 69 | 21 |
| 336 | Ogan Ilir | South Sumatra | 87 | 21 |
| 337 | Murung Raya | Central Kalimantan | 23 | 21 |
| 338 | Kota Sungai Penuh | Jambi | 21 | 21 |
| 339 | Kota Padang Sidempuan | North Sumatra | 47 | 21 |
| 340 | Tulang Bawang | Lampung | 88 | 21 |
| 341 | Bengkulu Selatan | Bengkulu | 35 | 21 |
| 342 | Banyuasin | South Sumatra | 168 | 21 |
| 343 | Bekasi | West Java | 550 | 21 |
| 344 | Tabalong | South Kalimantan | 50 | 21 |
| 345 | Batu Bara | North Sumatra | 76 | 20 |
| 346 | Mojokerto | East Java | 232 | 20 |
| 347 | Aceh Barat Daya | Aceh | 31 | 20 |
| 348 | Enrekang | South Sulawesi | 46 | 20 |
| 349 | Simeulue | Aceh | 19 | 20 |
| 350 | Alor | East Nusa Tenggara | 44 | 20 |
| 351 | Ogan Komering Ulu Selatan | South Sumatra | 84 | 20 |
| 352 | Kupang | East Nusa Tenggara | 76 | 20 |
| 353 | Biak Numfor | Papua | 29 | 20 |
| 354 | Halmahera Tengah | North Maluku | 11 | 20 |
| 355 | Sinjai | South Sulawesi | 52 | 20 |
| 356 | Sumba Barat | East Nusa Tenggara | 29 | 19 |
| 357 | Takalar | South Sulawesi | 56 | 19 |
| 358 | Muaro Jambi | Jambi | 70 | 19 |
| 359 | Halmahera Timur | North Maluku | 18 | 19 |
| 360 | Barito Kuala | South Kalimantan | 59 | 19 |
| 361 | Kota Tanjung Balai | North Sumatra | 33 | 19 |
| 362 | Belu | East Nusa Tenggara | 42 | 19 |
| 363 | Simalungun | North Sumatra | 191 | 18 |
| 364 | Kepahiang | Bengkulu | 28 | 18 |
| 365 | Majalengka | West Java | 241 | 18 |
| 366 | Pakpak Bharat | North Sumatra | 10 | 18 |
| 367 | Kota Gunungsitoli | North Sumatra | 25 | 18 |
| 368 | Kota Tebing Tinggi | North Sumatra | 32 | 18 |
| 369 | Kepulauan Sula | North Maluku | 19 | 18 |
| 370 | Kolaka | Southeast Sulawesi | 42 | 18 |
| 371 | Toba Samosir | North Sumatra | 38 | 18 |
| 372 | Aceh Utara | Aceh | 104 | 18 |
| 373 | Subang | West Java | 277 | 18 |
| 374 | Bandung | West Java | 623 | 17 |
| 375 | Dompu | West Nusa Tenggara | 39 | 17 |
| 376 | Kota Singkawang | West Kalimantan | 41 | 17 |
| 377 | Sumbawa Barat | West Nusa Tenggara | 24 | 17 |
| 378 | Sampang | East Java | 146 | 17 |
| 379 | Kota Bandung | West Java | 419 | 17 |
| 380 | Serang | Banten | 250 | 17 |
| 381 | Lebak | Banten | 217 | 17 |
| 382 | Bolaang Mongondow Selatan | North Sulawesi | 12 | 17 |
| 383 | Mimika | Papua | 52 | 17 |
| 384 | Luwu Timur | South Sulawesi | 50 | 17 |
| 385 | Wajo | South Sulawesi | 62 | 17 |
| 386 | Bandung Barat | West Java | 274 | 16 |
| 387 | Gowa | South Sulawesi | 124 | 16 |
| 388 | Manokwari Selatan | West Papua | 6 | 16 |
| 389 | Bombana | Southeast Sulawesi | 23 | 16 |
| 390 | Sumba Barat Daya | East Nusa Tenggara | 50 | 16 |
| 391 | Tasikmalaya | West Java | 285 | 16 |
| 392 | Tanjung Jabung Timur | Jambi | 36 | 16 |
| 393 | Way Kanan | Lampung | 75 | 16 |
| 394 | Muna | Southeast Sulawesi | 35 | 16 |
| 395 | Labuhanbatu | North Sumatra | 78 | 15 |
| 396 | Mamuju | West Sulawesi | 47 | 15 |
| 397 | Bantaeng | South Sulawesi | 31 | 15 |
| 398 | Kepulauan Aru | Maluku | 16 | 15 |
| 399 | Bolaang Mongondow | North Sulawesi | 37 | 15 |
| 400 | Jeneponto | South Sulawesi | 61 | 15 |
| 401 | Tangerang | Banten | 407 | 15 |
| 402 | Lombok Barat | West Nusa Tenggara | 103 | 14 |
| 403 | Tapanuli Tengah | North Sumatra | 51 | 14 |
| 404 | Bulukumba | South Sulawesi | 61 | 14 |
| 405 | Rejang Lebong | Bengkulu | 38 | 14 |
| 406 | Kubu Raya | West Kalimantan | 82 | 13 |
| 407 | Seruyan | Central Kalimantan | 20 | 13 |
| 408 | Maros | South Sulawesi | 52 | 13 |
| 409 | Sikka | East Nusa Tenggara | 43 | 13 |
| 410 | Ogan Komering Ilir | South Sumatra | 96 | 13 |
| 411 | Muna Barat | Southeast Sulawesi | 11 | 13 |
| 412 | Bener Meriah | Aceh | 21 | 13 |
| 413 | Malaka | East Nusa Tenggara | 25 | 13 |
| 414 | Sukabumi | West Java | 330 | 13 |
| 415 | Kepulauan Tanimbar | Maluku | 16 | 13 |
| 416 | Tambrauw | West Papua | 4 | 12 |
| 417 | Konawe Selatan | Southeast Sulawesi | 38 | 12 |
| 418 | Sumedang | West Java | 140 | 12 |
| 419 | Aceh Tenggara | Aceh | 51 | 12 |
| 420 | Labuhanbatu Utara | North Sumatra | 47 | 12 |
| 421 | Bolaang Mongondow Utara | North Sulawesi | 10 | 12 |
| 422 | Lombok Tengah | West Nusa Tenggara | 122 | 12 |
| 423 | Nagekeo | East Nusa Tenggara | 19 | 12 |
| 424 | Humbang Hasundutan | North Sumatra | 23 | 11 |
| 425 | Serdang Bedagai | North Sumatra | 75 | 11 |
| 426 | Maybrat | West Papua | 5 | 11 |
| 427 | Flores Timur | East Nusa Tenggara | 32 | 11 |
| 428 | Keerom | Papua | 7 | 11 |
| 429 | Maluku Tenggara | Maluku | 14 | 11 |
| 430 | Melawi | West Kalimantan | 25 | 11 |
| 431 | Empat Lawang | South Sumatra | 35 | 10 |
| 432 | Tapanuli Utara | North Sumatra | 33 | 10 |
| 433 | Adm. Kep. Seribu | Jakarta | 3 | 10 |
| 434 | Langkat | North Sumatra | 108 | 10 |
| 435 | Jayawijaya | Papua | 28 | 10 |
| 436 | Landak | West Kalimantan | 41 | 10 |
| 437 | Ketapang | West Kalimantan | 58 | 10 |
| 438 | Tana Toraja | South Sulawesi | 29 | 10 |
| 439 | Manggarai Timur | East Nusa Tenggara | 27 | 10 |
| 440 | Seram Bagian Barat | Maluku | 21 | 10 |
| 441 | Padang Lawas | North Sumatra | 26 | 10 |
| 442 | Barru | South Sulawesi | 18 | 10 |
| 443 | Halmahera Selatan | North Maluku | 24 | 10 |
| 444 | Kayong Utara | West Kalimantan | 12 | 9 |
| 445 | Timor Tengah Utara | East Nusa Tenggara | 25 | 9 |
| 446 | Bungo | Jambi | 32 | 9 |
| 447 | Karo | North Sumatra | 38 | 9 |
| 448 | Nabire | Papua | 16 | 9 |
| 449 | Buton Tengah | Southeast Sulawesi | 11 | 9 |
| 450 | Aceh Tengah | Aceh | 21 | 9 |
| 451 | Labuhanbatu Selatan | North Sumatra | 30 | 9 |
| 452 | Kaur | Bengkulu | 12 | 9 |
| 453 | Kerinci | Jambi | 22 | 9 |
| 454 | Cianjur | West Java | 208 | 9 |
| 455 | Tapanuli Selatan | North Sumatra | 28 | 9 |
| 456 | Mamasa | West Sulawesi | 17 | 8 |
| 457 | Kepulauan Talaud | North Sulawesi | 8 | 8 |
| 458 | Maluku Tengah | Maluku | 35 | 8 |
| 459 | Raja Ampat | West Papua | 5 | 8 |
| 460 | Seluma | Bengkulu | 16 | 8 |
| 461 | Lombok Utara | West Nusa Tenggara | 18 | 7 |
| 462 | Sidenreng Rappang | South Sulawesi | 23 | 7 |
| 463 | Buton Selatan | Southeast Sulawesi | 7 | 7 |
| 464 | Maluku Barat Daya | Maluku | 5 | 7 |
| 465 | Pulau Morotai | North Maluku | 5 | 7 |
| 466 | Kepulauan Yapen | Papua | 7 | 6 |
| 467 | Mandailing Natal | North Sumatra | 30 | 6 |
| 468 | Kapuas Hulu | West Kalimantan | 15 | 6 |
| 469 | Bima | West Nusa Tenggara | 31 | 6 |
| 470 | Kota Tual | Maluku | 5 | 6 |
| 471 | Sanggau | West Kalimantan | 26 | 5 |
| 472 | Mempawah | West Kalimantan | 16 | 5 |
| 473 | Padang Lawas Utara | North Sumatra | 14 | 5 |
| 474 | Sekadau | West Kalimantan | 11 | 5 |
| 475 | Timor Tengah Selatan | East Nusa Tenggara | 23 | 5 |
| 476 | Sambas | West Kalimantan | 31 | 5 |
| 477 | Bengkayang | West Kalimantan | 13 | 5 |
| 478 | Kepulauan Mentawai | West Sumatra | 4 | 5 |
| 479 | Bogor | West Java | 206 | 4 |
| 480 | Lombok Timur | West Nusa Tenggara | 48 | 4 |
| 481 | Buru | Maluku | 5 | 4 |
| 482 | Toraja Utara | South Sulawesi | 9 | 4 |
| 483 | Luwu | South Sulawesi | 12 | 3 |
| 484 | Sarolangun | Jambi | 9 | 3 |
| 485 | Konawe Kepulauan | Southeast Sulawesi | 1 | 3 |
| 486 | Nias | North Sumatra | 4 | 3 |
| 487 | Bone | South Sulawesi | 17 | 2 |
| 488 | Buru Selatan | Maluku | 1 | 1 |
| 489 | Nias Barat | North Sumatra | 1 | 1 |
| 490 | Mappi | Papua | 1 | 1 |
| 491 | Lebong | Bengkulu | 1 | 1 |
| 492 | Asmat | Papua | 1 | 1 |
| 493 | Paniai | Papua | 1 | 1 |
| 494 | Seram Bagian Timur | Maluku | 1 | 1 |
| 495 | Lanny Jaya | Papua | 1 | 0 |
| 496 | Deiyai | Papua | 0 | 0 |
| 497 | Toli Toli | Central Sulawesi | 0 | 0 |
| 498 | Nduga | Papua | 0 | 0 |
| 499 | Pegunungan Arfak | West Papua | 0 | 0 |
| 500 | Dogiyai | Papua | 0 | 0 |
| 501 | Sarmi | Papua | 0 | 0 |
| 502 | Waropen | Papua | 0 | 0 |
| 503 | Supiori | Papua | 0 | 0 |
| 504 | Puncak Jaya | Papua | 0 | 0 |
| 505 | Yahukimo | Papua | 0 | 0 |
| 506 | Mamberamo Raya | Papua | 0 | 0 |
| 507 | Yalimo | Papua | 0 | 0 |
| 508 | Intan Jaya | Papua | 0 | 0 |
| 509 | Pegunungan Bintang | Papua | 0 | 0 |
| 510 | Nias Selatan | North Sumatra | 0 | 0 |
| 511 | Mamberamo Tengah | Papua | 0 | 0 |
| 512 | Puncak | Papua | 0 | 0 |
| 513 | Nias Utara | North Sumatra | 0 | 0 |
| 514 | Pulau Taliabu | North Maluku | 0 | 0 |

**Supplementary Table 3. Summary of COVID-19 burden and other characteristics associated with higher COVID-19 mortality rate in Indonesia**

|  | **Mortality rate** | | | | |
| --- | --- | --- | --- | --- | --- |
|  | **Quartile 1**  **N (%)** | **Quartile 2 N (%)** | **Quartile 3 N (%)** | **Quartile 4 N (%)** | **p value** |
| COVID-19 mortality per 100,000 population |  |  |  |  |  |
| Quartile 1 (0.00–16.43) |  |  |  |  |  |
| Quartile 2 (16.43–31.47) |  |  |  |  |  |
| Quartile 3 (31.47–64.68) |  |  |  |  |  |
| Quartile 4 (64.68–283.82) |  |  |  |  |  |
| COVID-19 incidence per 100,000 population |  |  |  |  |  |
| Quartile 1 (8.13–604.91) | 83 (64.34) | 35 (27.34) | 10 (7.81) | 0 (0.00) | <0.001 |
| Quartile 2 (604.91–1063.79) | 30 (23.26) | 52 (40.63) | 38 (29.69) | 9 (6.98) |  |
| Quartile 3 (1063.79–1964.80) | 14 (10.85) | 35 (27.34) | 52 (40.63) | 28 (21.71) |  |
| Quartile 4 (1964.80–10,626.09) | 2 (1.55) | 6 (4.69) | 28 (21.88) | 92 (71.32) |  |
| Proportion of ≥60 years old population, % |  |  |  |  |  |
| Quartile 1 (2.01–7.22) | 53 (41.09) | 23 (17.97) | 31 (24.22) | 22 (17.05) | <0.001 |
| Quartile 2 (7.22–8.64) | 30 (23.26) | 49 (38.28) | 29 (22.66) | 20 (15.50) |  |
| Quartile 3 (8.64–10.43) | 27 (20.93) | 35 (27.34) | 36 (28.13) | 30 (23.26) |  |
| Quartile 4 (10.43–20.38) | 19 (14.73) | 21 (16.41) | 32 (25.00) | 57 (44.19) |  |
| Prevalence of hypertension, % |  |  |  |  |  |
| Quartile 1 (9.60–25.70) | 47 (36.43) | 44 (34.38) | 27 (21.09) | 12 (9.30) | <0.001 |
| Quartile 2 (25.70–30.30) | 35 (27.13) | 32 (25.00) | 35 (27.34) | 27 (20.93) |  |
| Quartile 3 (30.30–34.90) | 20 (15.50) | 34 (26.56) | 30 (23.44) | 46 (35.66) |  |
| Quartile 4 (34.90–50.00) | 27 (20.93) | 18 (14.06) | 36 (28.13) | 44 (34.11) |  |
| Prevalence of diabetes mellitus, % |  |  |  |  |  |
| Quartile 1 (0.00–1.10) | 64 (49.41) | 43 (33.59) | 19 (14.84) | 5 (3.88) | <0.001 |
| Quartile 2 (1.10–1.70) | 37 (28.68) | 36 (28.13) | 40 (31.25) | 23 (17.83) |  |
| Quartile 3 (1.70–2.50) | 20 (15.50) | 31 (24.22) | 41 (32.03) | 39 (30.23) |  |
| Quartile 4 (2.50–50.00) | 8 (6.20) | 18 (14.06) | 28 (21.88) | 62 (48.06) |  |
| Prevalence of central obesity, % |  |  |  |  |  |
| Quartile 1 (6.00–25.20) | 58 (44.96) | 36 (28.13) | 25 (19.53) | 10 (7.75) | <0.001 |
| Quartile 2 (25.20–30.40) | 23 (17.83) | 38 (29.69) | 40 (31.25) | 28 (21.71) |  |
| Quartile 3 (30.40–35.10) | 27 (20.93) | 31 (24.22) | 28 (21.88) | 42 (32.56) |  |
| Quartile 4 (35.10–50.00) | 21 (16.28) | 23 (17.97) | 35 (27.34) | 49 (37.98) |  |
| Prevalence of pneumonia, % |  |  |  |  |  |
| Quartile 1 (0.20–1.30) | 36 (27.91) | 34 (26.56) | 26 (20.31) | 44 (34.11) | <0.001 |
| Quartile 2 (1.30–1.80) | 31 (24.03) | 26 (20.31) | 36 (28.13) | 42 (32.56) |  |
| Quartile 3 (1.80–2.40) | 21 (16.28) | 33 (25.78) | 39 (30.47) | 25 (19.38) |  |
| Quartile 4 (2.40–13.00) | 41 (31.78) | 35 (27.34) | 27 (21.09) | 18 (13.95) |  |
| Vaccine coverage for ≥60 years old population, % |  |  |  |  |  |
| Quartile 1 (0.03–27.61) | 55 (42.64) | 30 (23.44) | 34 (26.56) | 10 (7.75) | <0.001 |
| Quartile 2 (27.61–36.52) | 31 (24.03) | 40 (31.25) | 35 (27.34) | 22 (17.05) |  |
| Quartile 3 (36.52–45.79) | 25 (19.38) | 37 (28.91) | 31 (24.22) | 36 (27.91) |  |
| Quartile 4 (45.79–86.20) | 18 (13.95) | 21 (16.41) | 28 (21.88) | 61 (47.29) |  |
| Doctor per 100,0000 population |  |  |  |  |  |
| Quartile 1 (0.15–5.43) | 34 (28.33) | 34 (26.98) | 40 (31.75) | 17 (13.28) | <0.001 |
| Quartile 2 (5.43–7.80) | 75 (62.50) | 83 (65.87) | 71 (56.35) | 90 (70.31) |  |
| Quartile 3 (7.80–11.74) | 7 (5.83) | 7 (5.56) | 13 (10.32) | 12 (9.38) |  |
| Quartile 4 (11.74–154.25) | 4 (3.33) | 2 (1.59) | 2 (1.59) | 9 (7.03) |  |
| Nurse per 100,0000 population |  |  |  |  |  |
| Quartile 1 (0.51–10.59) | 27 (22.50) | 24 (19.35) | 43 (33.59) | 31 (24.22) | <0.001 |
| Quartile 2 (10.59–17.95) | 27 (22.50) | 42 (33.87) | 29 (22.66) | 27 (21.09) |  |
| Quartile 3 (17.95–30.52) | 33 (27.50) | 32 (25.81) | 28 (21.88) | 32 (25.00) |  |
| Quartile 4 (30.52–238.28) | 33 (27.50) | 26 (20.97) | 28 (21.88) | 38 (29.69) |  |
| Midwife per 100,0000 population |  |  |  |  |  |
| Quartile 1 (0.28–11.46) | 45 (36.89) | 34 (27.20) | 30 (23.44) | 16 (12.50) | <0.001 |
| Quartile 2 (11.46–18.51) | 33 (27.05) | 32 (25.60) | 32 (25.00) | 30 (23.44) |  |
| Quartile 3 (18.51–27.13) | 26 (21.31) | 31 (24.80) | 32 (25.00) | 37 (28.91) |  |
| Quartile 4 (27.13–177.41) | 18 (14.75) | 28 (22.40) | 34 (26.56) | 45 (35.16) |  |
| Hospital per 100,0000 population |  |  |  |  |  |
| Quartile 1 (0.29–3.90) | 38 (36.19) | 35 (29.41) | 27 (21.77) | 19 (14.96) | <0.001 |
| Quartile 2 (3.90–7.39) | 31 (29.52) | 33 (27.73) | 34 (27.42) | 20 (15.75) |  |
| Quartile 3 (7.39–13.41) | 23 (21.90) | 34 (28.57) | 29 (23.39) | 33 (25.98) |  |
| Quartile 4 (13.41–135.21) | 13 (12.38) | 17 (14.29) | 34 (27.42) | 55 (43.31) |  |
| Expenditure, millions IDR |  |  |  |  |  |
| Quartile 1 (3.98–8.51) | 73 (56.59) | 29 (22.66) | 23 (17.97) | 4 (3.10) | <0.001 |
| Quartile 2 (8.51–10.11) | 29 (22.48) | 45 (35.16) | 36 (28.13) | 18 (13.95) |  |
| Quartile 3 (10.11–11.62) | 20 (15.50) | 39 (30.47) | 37 (28.91) | 33 (25.58) |  |
| Quartile 4 (11.62–23.58) | 7 (5.43) | 15 (11.72) | 32 (25.00) | 74 (57.36) |  |
| Life expectancy, years |  |  |  |  |  |
| Quartile 1 (55.27–67.24) | 61 (47.29) | 40 (31.25) | 24 (18.75) | 5 (3.88) | <0.001 |
| Quartile 2 (67.24–69.91) | 41 (31.78) | 44 (34.38) | 35 (27.34) | 7 (5.43) |  |
| Quartile 3 (69.91–71.87) | 19 (14.73) | 31 (24.22) | 41 (32.03) | 38 (29.46) |  |
| Quartile 4 (71.87–77.65) | 8 (6.20) | 13 (10.16) | 28 (21.88) | 79 (61.24) |  |
| Mean length of formal education, years |  |  |  |  |  |
| Quartile 1 (1.13–7.43) | 56 (43.41) | 24 (18.75) | 29 (22.66) | 20 (15.50) | <0.001 |
| Quartile 2 (7.43–8.24) | 31 (24.03) | 40 (31.25) | 35 (27.34) | 24 (18.60) |  |
| Quartile 3 (8.24–9.28) | 28 (21.71) | 45 (35.16) | 27 (21.09) | 27 (20.93) |  |
| Quartile 4 (9.28–12.65) | 14 (10.85) | 19 (14.84) | 37 (28.91) | 58 (44.96) |  |

**Supplementary Table 4. Correlation matrix between each district-level variable assessed in this study**

|  | Mortality rate | Incidence rate | Elder population | Hypertension | Diabetes mellitus | Obesity | Pneumonia | Vaccine coverage | Doctor ratio | Nurse ratio | Midwife ratio | Hospital ratio | Expenditure | Life expectancy | Education |
| --- | --- | --- | --- | --- | --- | --- | --- | --- | --- | --- | --- | --- | --- | --- | --- |
| Mortality rate |  |  |  |  |  |  |  |  |  |  |  |  |  |  |  |
| Incidence rate | 0.0000 |  |  |  |  |  |  |  |  |  |  |  |  |  |  |
| Elder population | 0.0000 | 0.0169 |  |  |  |  |  |  |  |  |  |  |  |  |  |
| Hypertension | 0.0000 | 0.0000 | 0.0000 |  |  |  |  |  |  |  |  |  |  |  |  |
| Diabetes mellitus | 0.0000 | 0.0000 | 0.0000 | 0.0007 |  |  |  |  |  |  |  |  |  |  |  |
| Obesity | 0.0000 | 0.0000 | 0.0307 | 0.8458 | 0.0000 |  |  |  |  |  |  |  |  |  |  |
| Pneumonia | 0.0316 | 0.0179 | 0.0001 | 0.8889 | 0.2477 | 0.2400 |  |  |  |  |  |  |  |  |  |
| Vaccine coverage | 0.0000 | 0.0000 | 0.0000 | 0.0000 | 0.0000 | 0.0000 | 0.0000 |  |  |  |  |  |  |  |  |
| Doctor ratio | 0.0147 | 0.0000 | 0.0019 | 0.0007 | 0.0010 | 0.0010 | 0.5070 | 0.4155 |  |  |  |  |  |  |  |
| Nurse ratio | 0.7569 | 0.0000 | 0.0000 | 0.0001 | 0.6374 | 0.0255 | 0.1832 | 0.0003 | 0.0000 |  |  |  |  |  |  |
| Midwife ratio | 0.0000 | 0.0000 | 0.4821 | 0.6588 | 0.2270 | 0.3261 | 0.1593 | 0.0000 | 0.0000 | 0.0005 |  |  |  |  |  |
| Hospital ratio | 0.0000 | 0.0000 | 0.3757 | 0.0342 | 0.0000 | 0.0000 | 0.5500 | 0.0000 | 0.0000 | 0.0000 | 0.0000 |  |  |  |  |
| Expenditure | 0.0000 | 0.0000 | 0.0007 | 0.0010 | 0.0000 | 0.0000 | 0.0027 | 0.0000 | 0.0045 | 0.8661 | 0.1863 | 0.0000 |  |  |  |
| Life expectancy | 0.0000 | 0.0000 | 0.0000 | 0.0000 | 0.0000 | 0.0000 | 0.2332 | 0.0000 | 0.2701 | 0.0003 | 0.0032 | 0.0000 | 0.0000 |  |  |
| Education | 0.0000 | 0.0000 | 0.0384 | 0.0020 | 0.0000 | 0.0000 | 0.7313 | 0.0000 | 0.0000 | 0.0000 | 0.0002 | 0.0000 | 0.0000 | 0.0000 |  |
